# Inhibition of CEMIP potentiates the effect of sorafenib on metastatic hepatocellular carcinoma by reducing the stiffness of lung metastases

**DOI:** 10.1038/s41419-023-05550-4

**Published:** 2023-01-13

**Authors:** Mingyu Liu, Lulu Xie, Yuying Zhang, Jianning Chen, Xiang Zhang, Ye Chen, Wensou Huang, Mingyue Cai, Licong Liang, Miaoling Lai, Jingjun Huang, Yongjian Guo, Liteng Lin, Kangshun Zhu

**Affiliations:** 1 Laboratory of Interventional Radiology, Department of Minimally Invasive Interventional Radiology, and Department of Radiology, the Second Affiliated Hospital of Guangzhou Medical University, 510260 Guangzhou, Guangdong China; 2 Central Laboratory, Shenzhen Longhua Maternity and Child Healthcare Hospital, 518109 Shenzhen, China; 3 Department of Pathology, The Third Affiliated Hospital of Sun Yat-Sen University, 510630 Guangzhou, China; 4 State Key Laboratory of Digestive Disease, Institute of Digestive Disease and The Department of Medicine and Therapeutics, Li Ka Shing Institute of Health Sciences, CUHK Shenzhen Research Institute, The Chinese University of Hong Kong, Hong Kong SAR, China; 5 Department of Pathology, the Second Affiliated Hospital of Guangzhou Medical University, 510260 Guangzhou, Guangdong China

**Keywords:** Molecular biology, Pathogenesis

## Abstract

Hepatocellular carcinoma (HCC) with lung metastasis is associated with poor prognosis and poor therapeutic outcomes. Studies have demonstrated that stiffened stroma can promote metastasis in various tumors. However, how the lung mechanical microenvironment favors circulating tumor cells remains unclear in metastatic HCC. Here, we found that the expression of cell migration-inducing hyaluronan-binding protein (CEMIP) was closely associated with lung metastasis and can promote pre-metastatic niche formation by increasing lung matrix stiffness. Furthermore, upregulated serum CEMIP was indicative of lung fibrotic changes severity in patients with HCC lung metastasis. By directly targeting CEMIP, pirfenidone can inhibit CEMIP/TGF-β1/Smad signaling pathway and reduce lung metastases stiffening, demonstrating promising antitumor activity. Pirfenidone in combination with sorafenib can more effectively suppress the incidence of lung metastasis compared with sorafenib alone. This study is the first attempt to modulate the mechanical microenvironment for HCC therapy and highlights CEMIP as a potential target for the prevention and treatment of HCC lung metastasis.

**Figure:**
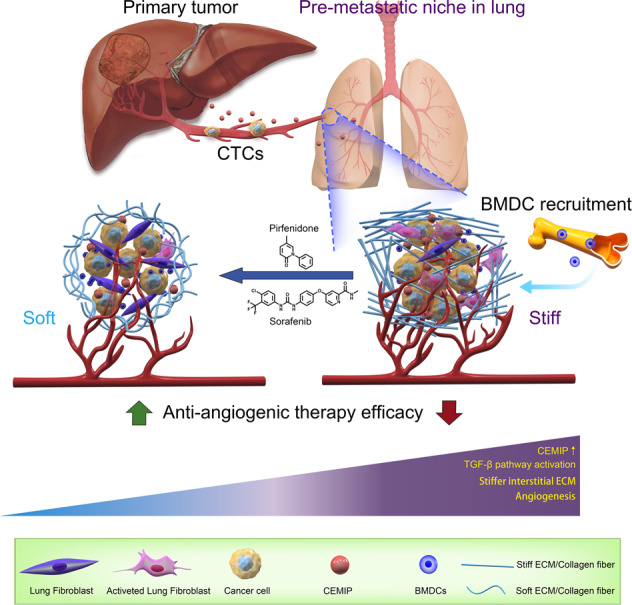
CEMIP mediating an HCC-permissive microenvironment through controlling matrix stiffness. Meanwhile, Pirfenidone could reduce metastasis stiffness and increases the anti-angiogenic effect of Sorafenib by directly targeting CEMIP.

CEMIP mediating an HCC-permissive microenvironment through controlling matrix stiffness. Meanwhile, Pirfenidone could reduce metastasis stiffness and increases the anti-angiogenic effect of Sorafenib by directly targeting CEMIP.

## Introduction

Hepatocellular carcinoma (HCC) has a poor prognosis due to its rapid development and early metastasis [1]. Extrahepatic metastasis, especially lung metastasis, has become the key issue that affects the long-term survival of patients. Sorafenib is used as first-line systemic therapy for advanced HCC. However, for patients with extrahepatic metastasis, the therapeutic effect is limited and the median progression-free survival (PFS) is only 4.0 months [2]. Even the promising atezolizumab plus bevacizumab therapy could only increase the median PFS to 6.8 months in patients with unresectable hepatocellular carcinoma [3]. Therefore, it is imperative to further investigate the molecular mechanism of lung metastasis and explore effective therapy options in HCC patients with lung metastases.

The formation of the pre-metastatic niche has been proven to be a prerequisite for metastasis to distant organs [4]. Extracellular matrix (ECM) is a noncellular three-dimensional network composed of collagens, fibronectin, and several other glycoproteins [5]. Accumulating evidence suggests that ECM stiffening plays a vital role in the formation of the pre-metastatic niche, as it results in the generation of a broad range of biophysical and biochemical stimuli for tumor angiogenesis, epithelial-to-mesenchymal transition (EMT), invasion, migration, etc. [6–8]. As the most abundant cell type in the tumor ECM, fibroblasts, especially those at the metastatic site, may trans-differentiate into an activated phenotype and play a key role in modifying the metastatic microenvironment in distant organs [9, 10]. Encouragingly, as the role of tumorigenic ECM stiffening is becoming clearer, novel ECM-targeted therapies are gaining increased attention [11]. However, to date, the exact role of ECM stiffness in HCC remains unclear, and is even more obscure in HCC patients with lung metastasis.

Cell migration-inducing and hyaluronan-binding protein (CEMIP, also known as KIAA1199) is a secreted protein containing two GG domains and a special G8 domain that plays a crucial role in hyaluronic acid degradation and cellular proliferation [12, 13]. Although it was originally discovered as a regulator of non-syndromic hearing loss [14], CEMIP has been recognized to be crucial for invasion and distant metastasis in various tumors [15–17]. In HCC, CEMIP has also been found to promote sorafenib tolerance via EGF/EGFR-dependent EMT [18]. Notably, a recent study has demonstrated a potent effect of CEMIP on the aggravation of lung fibrosis by stimulating ECM production and stiffness [19]. However, the role of CEMIP in the regulation of ECM stiffness during the process of HCC lung metastasis has not been reported.

In this study, we elucidate that the HCC-secreted CEMIP protein activates the lung fibroblasts (LFs) to mediate lung ECM stiffening, which facilitates the formation of a pre-metastatic niche that promotes HCC lung metastasis. Our findings personalize metastatic HCC therapy with a mechano-predictive biomarker and could serve as a foundation for the application of mechanotherapeutics in clinical practice.

## Materials and methods

### Atomic force microscopy measurements

Atomic force microscopy (AFM, Dimension FastScan, Bruker, Germany) was used to measure the Young’s elastic modulus of the lung stroma in different groups. Frozen lung samples from humans and mice were sliced into pieces of 50 μm in thickness. Each slide was immersed in PBS and was then anchored to the stage of the microscope.

The stiffness values of different groups of lung tissue were analyzed for comparison. Young’s modulus was taken as the average of measurements from three random locations. A tipless silicon probe (Bruker MLCT O10) was attached to a PS sphere with a diameter of 5 μm, and the calibrated spring constant was 0.07 N/m. This preparative tip was used to analyze tissue samples. Force measurements were collected over a 60 × 60-μm grid. The resulting force data were converted to elastic modulus values according to the HERTZ model.

### Polyacrylamide hydrogels

Commercially available polyacrylamide hydrogels polystyrene plates of various stiffness coated with type I collagen were used (Softwell; Matrigen Life Technologies, Brea, CA). Polyacrylamide gels with stiffnesses of 2 kPa and 25 kPa were chosen to represent the stiffness of normal and fibrotic lungs, respectively, based on previously published data [20].

### Detection of pulmonary function

MasterSceen (Germany) pulmonary function instrument was used by specialized technicians to determine pulmonary function in all participants. People who never smokes, or stopped smoking at least 3 years before recruitment were enrolled. Lung function was determined by forced expiratory volume in 1 s (FEV1), forced vital capacity (FVC), and diffusion capacity of carbon monoxide (DLCO), that used as an indirect marker for interstitial lung change. All indicators are expressed as the percentage of the normal predicted value (based on the actual age, height, and weight).

### Statistical analysis

All analyses were performed using GraphPad Prism software (GraphPad Software Inc. version 8.0). The experiment data are presented as mean ± SEM. Differences between two groups or multiple groups were analyzed by Student’s *t* test and ANOVA, respectively. Pearson’s correlation was used to detect and analyze the correlation between CEMIP concentration and each pulmonary function parameter in patients with HCC. Data are reported, including estimation of variation within each group. *P* values <0.05 were considered significant.

Other detailed materials and methods are described in Supplementary Information.

## Results

### Clinical significance of CEMIP expression in HCC

**Fig. 1:**
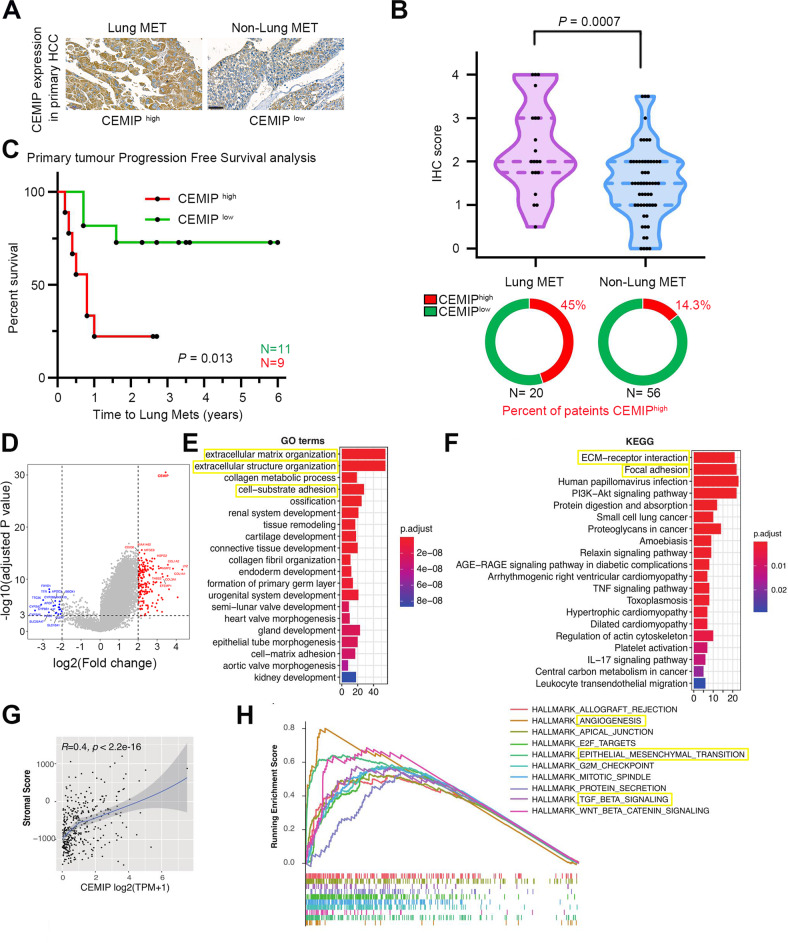
The CEMIP expression level is associated with lung metastatic progression in liver cancer. **A** Representative immunohistochemistry images illustrating CEMIP expression levels in patients with or without lung metastasis. Scale bars, 50 μm. **B** CEMIP immunohistochemistry staining scores in primary HCC tissues obtained from patients with or without lung metastasis. The CEMIP expression level was significantly higher in the pulmonary metastatic group than in the non-metastatic group (*P* < 0.05). **C** Kaplan–Meier curves depicting the time to first lung metastasis (from diagnosis time) based on whether CEMIP expression levels in the primary tumor were low (green) or high (red). **D** Volcano plot showing the differentially expressed genes (DEGs) between samples exhibiting high (top 10%) and low (bottom 10%) levels of CEMIP expression in the TCGA cohort. **E**, **F** GO and KEGG enrichment analyses of significant DEGs (Log2|fold change | >2 and *P* < 0.001). **G** Scatter plots depicting the Spearman correlations for CEMIP expression and stromal score. **H** Gene set enrichment analysis (GSEA) plot showing the enrichment of tumor hallmarks in tumors exhibiting high levels of CEMIP expression (top 10%), in comparison to that in tumors exhibiting low levels of CEMIP expression (bottom 10%).

To determine the correlation between CEMIP expression levels and clinicopathological features, we first characterized CEMIP expression via immunohistochemistry analysis of paraffin sections of liver tissues obtained from the primary tumors (PTs) of HCC patients with lung metastases (20 samples) or no metastases (56 samples). Based on the staining intensity, tissues were categorized into groups with low (staining score 0–2) and high (staining score >2–4) scores (Supplementary Fig. S1A). As shown in (Fig. 1A, B), patients with lung metastases exhibited a significantly higher level of CEMIP expression than those without metastasis. Notably, 45% (9/20) of patients with lung metastases were detected to have high CEMIP expression, compared to 14.3% (8/56) in those without lung metastases (*P* = 0.005) (Fig. 1B). Furthermore, high CEMIP expression levels in PTs were correlated with a shorter latency period for lung metastasis (Fig. 1C). Therefore, high CEMIP expression in surgically resected HCC specimens might be suggestive of lung metastatic risk at the early stages.

Next, we performed GO and KEGG enrichment analysis of differentially expressed genes (DEGs) between samples exhibiting high (top 10%) and low (bottom 10%) levels of CEMIP expression using the TCGA HCC dataset. As shown (in Fig. 1D, E), the DEGs that were predominantly upregulated in the CEMIP-high subgroup were substantially enriched in gene sets involved in the generation of stromal tissues in tumors, including in “extracellular matrix organization”, “extracellular structure organization”, “cell–substrate adhesion”, etc. Consistently, KEGG pathway enrichment also revealed a predominant enrichment of the pathways involved in “ECM–receptor interactions” and “focal adhesion” (Fig. 1F). Further, a significant positive correlation between the stromal activity and CEMIP expression was observed (Fig. 1G). The GSEA results also revealed the activation of tumor hallmarks associated with high CEMIP expression levels, i.e., angiogenesis, EMT, and TGF-beta overexpression (Fig. 1H). Taken together, these findings suggested a crucial role of CEMIP in shaping stromal tissues in tumors, which may facilitate metastases.

### CEMIP remodels the ECM and promotes lung pre-metastatic niche formation

**Fig. 2:**
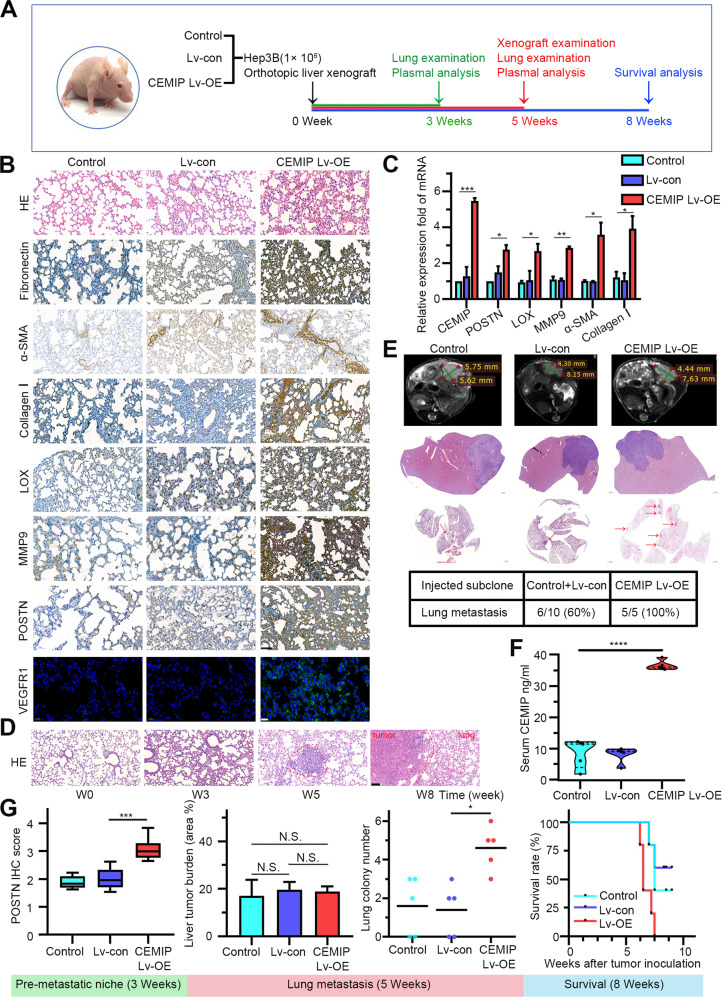
CEMIP modulates lung pre-metastatic niche formation to support metastasis. **A** Schematic of the animal experiments conducted using BALB/c nude mice. **B** Immunohistochemistry staining of FN, α-SMA, LOX, MMP9, collagen I, and POSTN in lung sections of 3-week-old mice injected with the CEMIP overexpressing lentivirus and control lentivirus. The untreated group was used as a negative control. Scale bar = 50 μm. Immunofluorescent staining of VEGFR1 on lung paraffin sections. Scale bar = 20 μm. **C** Niche gene expression was detected in the lung sections of 3-week-old mice via qRT-PCR analysis. **D** Representative hematoxylin–eosin-staining stained images of the mouse lung in the CEMIP-overexpressing lentivirus group at different time points. Scale bar = 100 μm. **E** Liver MR was performed for the detection of the hepatic mass. H&E staining of orthotopic liver tumors and lung foci in mice exhibiting lung metastasis development. The incidence of lung metastasis development in mice injected with Hep3B subclones was shown in the table. **F** Detection of plasma CEMIP expression levels in 5-week-old mice. The CEMIP expression level was significantly higher in the CEMIP-overexpressing group (*P* < 0.0001 by one-way ANOVA). The horizontal lines represent the median values. **G** POSTN IHC score analysis in the lungs of mice at week 3. Liver tumor burden and lung metastatic colony number analyses of mice at week 5. Survival analysis of mice raised for up to 8 weeks.

To obtain an overall view of the role of CEMIP in the lung metastatic process, we performed an orthotopic transplantation experiment, by injecting male BALB/c nude mice with 1 × 10^6^ control, or CEMIP-overexpressing Hep3B subclones. Mice were subjected to orthotopic liver implantation and raised for 3, 5, or 8 weeks (Fig. 2A). After 3 weeks, no metastasis was found in the lungs, but the expression levels of niche characteristic genes [21], including *fibronectin* (*FN*), *MMP9*, and *LOX* were higher in the CEMIP overexpressing group. POSTN, a protein that has been identified as a key player involved in the priming of the lung metastatic niche and strengthening of the CTCs settled onto the remodeled matrix “soil” [22], was also considerably upregulated. Most notably, a significantly higher expression of α-SMA and COL-I was observed in the CEMIP overexpression group, which was indicative of the ECM remodeling features of CEMIP (Fig. 2B).

Primary tumor-secreted soluble molecules, bone-marrow-derived cells (BMDCs) recruitment, and the local stromal microenvironment change are the three major factors crucial for the formation of pre-metastatic niche [23]. Hematopoietic progenitor cells (HPCs) that express VEGF receptor-1 (VEGFR1) may be mobilized and recruited from bone marrow to form distinct cell clusters in secondary sites before and during pre-metastatic niche formation. Meanwhile, these VEGFR1 + HPCs mediate their recruitment into fibronectin-rich local microenvironments where the pre-metastatic niche is set up [24]. Our immunofluorescence staining results suggested that treatment with CEMIP-overexpressing lentivirus increased the number of VEGFR1-positive cells in the lungs (Fig. 2B). To visualize the expression patterns more clearly, we quantified the expression of VEGFR1 in lung tissue across different time periods (Supplementary Fig. S1B). Quantitative reverse-transcriptase PCR (qRT-PCR) analysis further confirmed that the expression of niche characteristic genes was elevated in the lungs of mice from the CEMIP-overexpressing group (Fig. 2C). These results suggested that CEMIP increase the initial seeding and play a role in pre-metastatic niche formation.

Lung tissues were harvested and examined at different time points. Fibrotic changes were visible in the lungs 3 weeks after CEMIP induction (Fig. 2D). In the 5th week, livers and lungs were examined. Results show more abundant lung metastatic colonies in the CEMIP overexpressing group compared to the control. While no significant differences in liver tumor burden were observed between groups, indicating that CEMIP-mediated lung metastasis is independent of primary liver tumor expansion (Fig. 2E, G). Meanwhile, plasma levels of CEMIP were also significantly higher in the CEMIP overexpressing group (Fig. 2F). Moreover, mice with CEMIP overexpressing lentivirus injection had shorter survival times than mice of the control group followed up to 8 weeks, (Fig. 2G). Taken together, our data suggested that CEMIP can promote pre-metastatic niche formation and ECM remodeling at an early stage, leading to lung metastasis and poor prognosis.

### CEMIP activates lung fibroblasts and facilitates tumor cell metastatic colonization

**Fig. 3:**
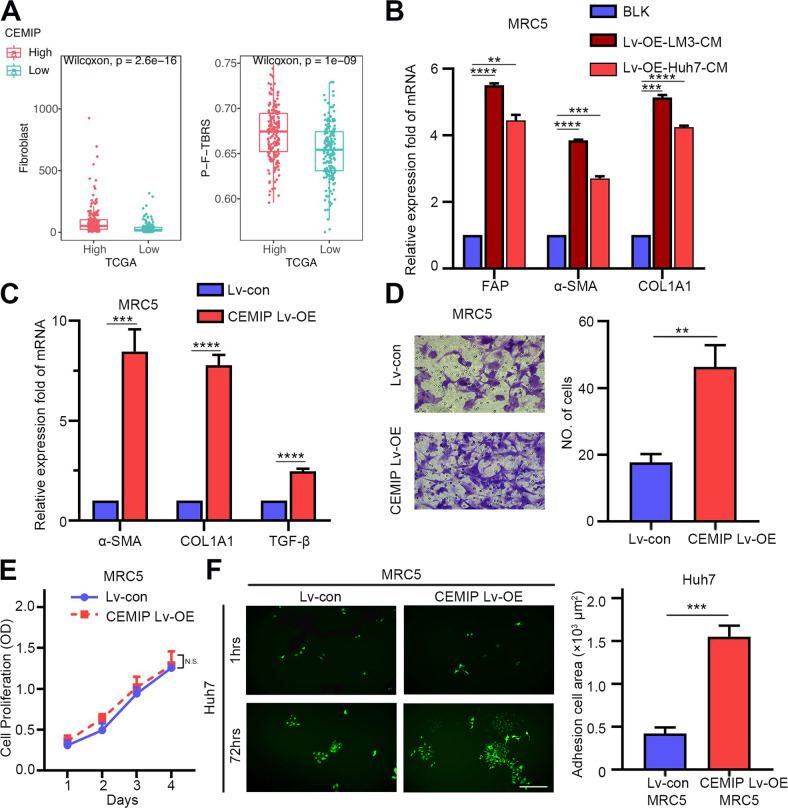
CEMIP regulates lung fibroblast activation to promote liver cancer progression. **A** The association between CEMIP and fibroblasts. A comparison of the extent of fibroblast infiltration in the tumor microenvironment between tumors exhibiting high and low levels of CEMIP expression (left panel). A comparison of the pan-fibroblast TGF-beta signature scores between tumors exhibiting high and low levels of CEMIP expression (right panel). **B** Indicated fibroblast marker expression levels in MRC5 cells treated with CM from CEMIP-overexpressing lentivirus-transfected HCC cells were detected by qRT-PCR analysis. **C** The expression levels of α-SMA, COL1A1, and TGF-β in MRC5 cells treated with the CEMIP-overexpressing lentivirus or blank control were detected by qRT-PCR analysis. **D** Migration assays of MRC5 cells treated with the CEMIP-overexpressing lentivirus or blank control. Migrated cells were counted, and representative images are shown. **E** MRC5 cells were stably infected with the empty vector or CEMIP-overexpressing lentivirus and cultured for 1–4 days. Cell proliferation was detected by the Cell Counting Kit-8 (CCK-8) assay. **F** Images and quantification of adhesion and proliferation of Huh7-GFP cells (green) on LFs (MRC5) treated with the CEMIP-overexpressing lentivirus or blank control. Scale bar = 100 μm.

Fibroblasts are the major stromal cells surrounding tumors and are crucial for tumor metastases and treatment resistance [25]. In this study, fibroblast levels determined by the “MCPcounter” algorithm were elevated in samples with high CEMIP expression levels. Moreover, the pan-fibroblast-TGF-beta signature score was significantly higher in samples with high levels of CEMIP, compared to their counterparts. Thus, we conclude that CEMIP is associated with the activation of the TGF-beta signaling pathway in fibroblasts (Fig. 3A).

Activated fibroblasts in the metastatic niche have been demonstrated to actively participate in tumor metastasis progression [26]. Considering the importance of intercellular communication between tumor cells and the surrounding microenvironment, we then evaluated whether CEMIP serves as a functional factor secreted by HCCs that affects the features of MRC5 human lung fibroblasts. After being transfected with CEMIP-overexpressing lentivirus, the conditioned media (CM) of LM3 and Huh7 cells were collected and used for the incubation of MRC5 cells for 48 h. The results revealed that the incubation of MRC5 cells with CM containing overexpressed CEMIP resulted in higher levels of fibroblast markers, including fibroblast activation protein (FAP), α-SMA, and collagen type I (COL1A1) (Fig. 3B). Consistently, when MRC5 cells were directly transfected with CEMIP-overexpressing lentivirus, α-SMA, COL1A1, and TGF-β were also observed to be significantly upregulated (Fig. 3C). In addition, the extent of fibroblasts migration in the CEMIP-overexpressing group was higher, compared to that of the control group (Fig. 3D). Interestingly, CEMIP overexpression did not affect the proliferation of MRC5 cells (Fig. 3E), suggesting that CEMIP promoted cell migration in a cell proliferation-independent manner. Altogether, our data suggest that CEMIP can activate LFs to facilitate tumor metastases.

To evaluate the tumor permissive ability of LFs treated with CEMIP, Huh7-GFP cells were added to wells with pre-cultured MRC5 with or without CEMIP overexpression. The results demonstrated that more tumor cells adhered to LFs with CEMIP overexpression compared to their counterpart. When these adhesive HCC cells were cultured for another 72 h, tumor colonies were formed. The tumor colony area was significantly larger in the CEMIP-overexpressing group than in the control group (Fig. 3F). Taken together, these findings indicated that CEMIP improved the tumor permissive abilities of LFs, in terms of tumor cell adhesion and colony formation.

### CEMIP increases lung matrix hardness and promotes metastasis in a stiffness-dependent manner

**Fig. 4:**
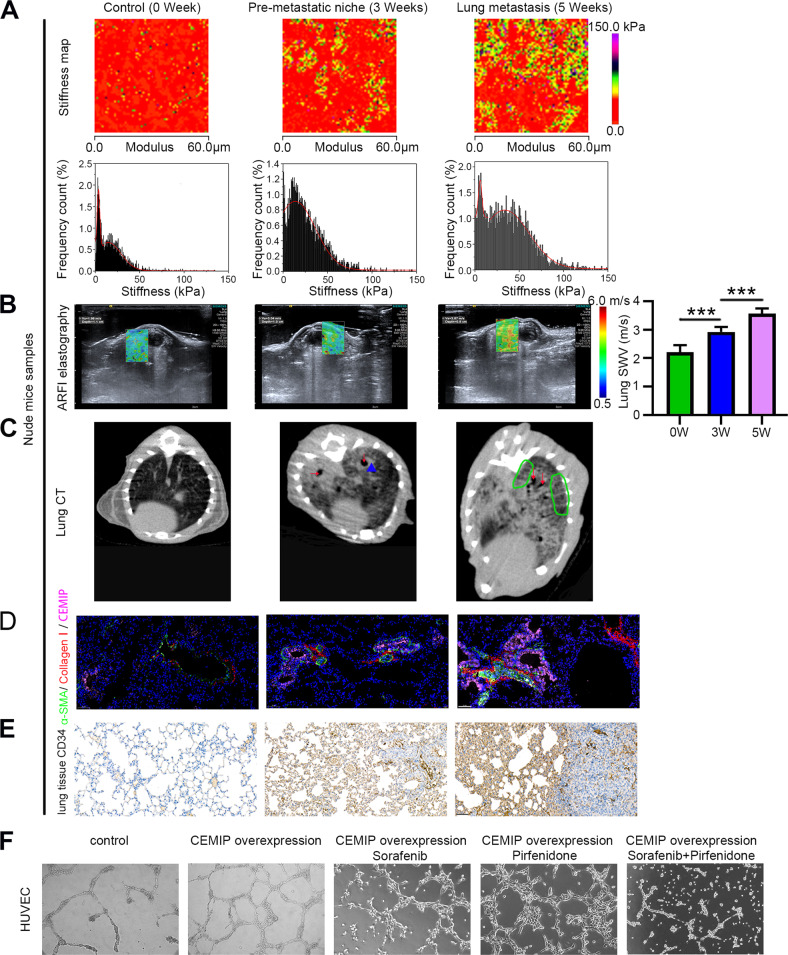
CEMIP promotes angiogenesis by increasing ECM stiffness. **A** Stiffness maps and stiffness distribution of lung tissues were measured using AFM. Lung stiffness was significantly elevated in the 3-week-pre-metastatic niche and was highest in 5-week-lung metastases. **B** Comparison of wave speeds among 0-week, 3-week, and 5-week groups via SWE measurement ***P < 0.001. **C** Left: Normal CT appearance. Middle: The predominant abnormality is patchy and exhibits bilateral ground-glass opacification, peribronchial consolidation (blue triangle), and tractional bronchiectasis (red arrows), and its appearance was consistent with that of fibrosis. Right: Sagittal reformation shows numerous thickened interlobular septa (green circle), peripheral or peribronchial consolidation, and mild reticular changes representative of typical lung fibrosis. **D** Immunofluorescent staining of COL-I (red), α-SMA (green), and CEMIP (pink) on paraffin-processed lung tissue sections. Scale bar = 50 μm. **E** CD34 staining of paraffin sections of lung tissues obtained from nude mice. Scale bar = 50 μm. **F** Representative images of tube formation in HUVECs cultured on Matrigel-coated plates with CEMIP-overexpressing lentivirus treated with pirfenidone/sorafenib.

Highly activated metastasis-associated fibroblasts lead to ECM stiffening [9] and matrix stiffening is known to affect microvessel outgrowth and branching [27]. By using atomic force microscopy (AFM), we then probe the regulatory effects of ECM stiffening on the lung metastatic potential. At first, we found that CEMIP can significantly increase lung stromal stiffness. In addition, there is a strong correlation between increased tissue stiffness and tumor growth at the lung metastatic site (Supplementary Fig. S1C). Also in the established nude mice xenograft model, we next demonstrated that metastases resulted in increased lung stromal stiffness compared to the pre-metastatic niche while non-metastatic lung tissues showed the lowest level of stiffness (Fig. 4A). We then used shear wave elastography (SWE), a new ultrasound-based elastography technique, to semi-quantitatively measure lung parenchymal stiffness without invasiveness. The results of SWE analysis indicated that the average shear wave velocity (SWV) of the 5-week-old lung metastasis group (3.57 m/s) was significantly higher than that of the 3-week-old pre-metastatic niches group (2.92 m/s) and 0-week-old non-metastasis group (2.21 m/s) (Fig. 4B). Moreover, thoracic CT in the 5-week group revealed typical radiographic features associated with lung fibrosis―tractional bronchiectasis and reticular changes (Fig. 4C). Immunofluorescence staining analysis also proved that stiffness was correlated with COL-I, α-SMA, and CEMIP expression in samples from the same mouse (Fig. 4D). Furthermore, a positive correlation between lung stiffness and CD34 + cells within the lung stroma was observed in the nude mice xenograft model (Fig. 4E). Together, these findings demonstrate CEMIP-mediated Matrix Stiffening can increase metastasis and promote aberrant tumor vasculature during HCC progression.

We further validated the mechanical cues in the microenvironment using human paraffinized tissue specimens. We observed a significant increase in the stromal stiffness in lung para-metastases of HCC patients, compared to that in the benign lung lesion samples (obtained via pulmonary bullectomy) (Supplementary Fig. S2A). In representative cases of metastatic HCC samples, immunohistochemistry results also corroborate the role of CEMIP in mediating tumor-associated neovascularization (Supplementary Fig. S2B).

Next, we evaluated the effects of CEMIP-induced matrix stiffness on various malignant properties in vitro using polyacrylamide hydrogels with different levels of stiffness. Polyacrylamide gels with the stiffness of 2 kPa and 25 kPa were chosen to represent the stiffness of the normal and fibrotic lung, respectively [28, 29]. Results from CCK-8 assay, q-PCR, EdU and transwell assay revealed that ECM stiffening can promote tumor growth (Supplementary Fig. S1D–F). On the other hand, CEMIP-induced metastatic event is dependent on matrix stiffness (Supplementary Fig. S1G), while CEMIP on its own did not exert a significant impact on cell proliferation (Supplementary Fig. S1F). To further evaluate its effect on vascular morphogenesis quantitatively, we performed a tubule formation assay using matrigels (Fig. 4F) and polyacrylamide gels with different stiffness (Supplementary Fig. S1H). The results demonstrated that CEMIP showed a strong pro-angiogenic effect and increased loop formation on stiffer substrates (25 kPa gel). Overall, CEMIP significantly increased cell migration and angiogenesis in a stiffness-dependent manner. Therefore, we infer that CEMIP drives HCC progression under conditions of a fibrotic/stiff lung environment.

### Pirfenidone reduces lung metastasis stiffness by inhibiting the CEMIP/TGF–β1/Smad signaling pathway and increases the anti-angiogenic therapeutic effect of sorafenib

**Fig. 5:**
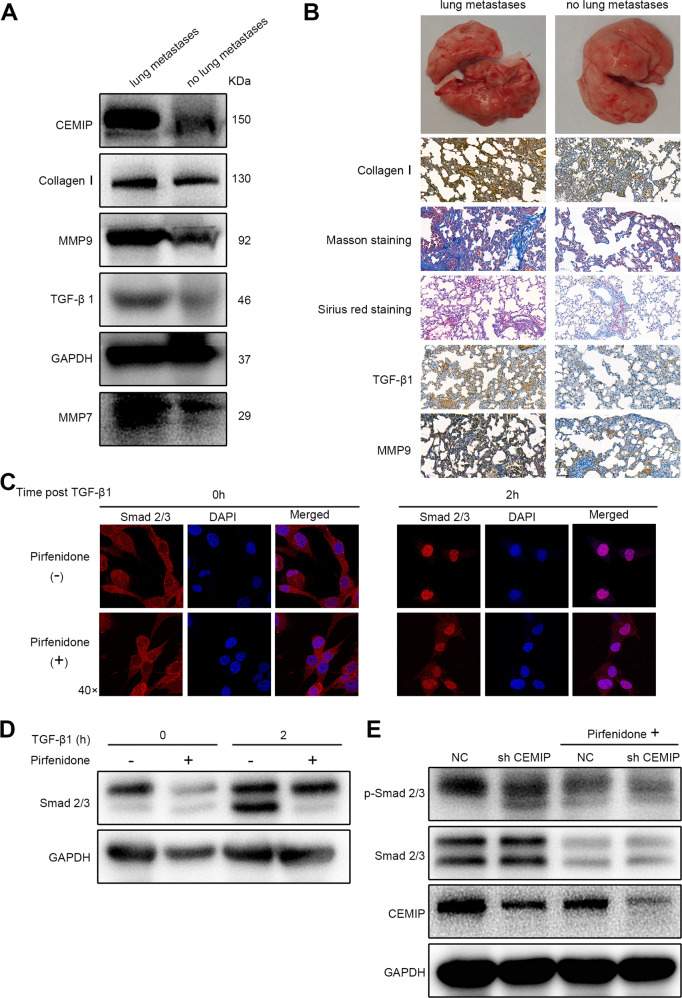
The pirfenidone-induced reduction of CEMIP may inhibit the canonical TGF-β-Smad signaling. **A**, **B** The evaluation of lung tissues via western blotting and IHC proved that matrix stiffening was observed in mice with or without lung metastasis. Scale bar = 50 μm. **C** MRC5 fibroblasts were treated with or without 2 mg/mL pirfenidone for a total of 12 h and 10 ng/mL TGF-β1 for 2 h. Representative immunofluorescence images demonstrate the nuclear accumulation of Smad2 reduced after pirfenidone treatment (magnification ×40). **D** MRC5 fibroblasts were treated with 2 mg/mL pirfenidone for a total of 12 h and 10 ng/mL TGF-β1 for 2 h. Protein lysates were analyzed via western blotting. Pirfenidone treatment resulted in a reduction in Smad2 and Smad3 post-TGF-β1 treatment. **E** The inhibitory effects of pirfenidone on the CEMIP-induced expression of p-Smad2/3 in MRC5 fibroblasts.

Studies have shown that the TGF-β signaling pathway is involved in the activation of fibroblasts which resulted in an increase in ECM stiffness and modulates tissue remodeling marker MMPs [30–33]. Here, we proved that CEMIP-mediated matrix stiffness could promote the secretion of autocrine TGF-β1 from LFs (Supplementary Fig. S3A). Next, in the in vivo nude mouse orthotopic transplantation model, proteins were extracted from lung tissues. Western blot analysis showed an enhanced expression of CEMIP, MMP7, MMP9, and TGF-β1 in individuals with lung metastasis compared to those without lung metastases (Fig. 5A). Masson’s trichrome and Sirius red stains showed lung fibrosis-like changes in mice with lung metastases (Fig. 5B). Altogether, our results suggested an indispensable role of TGF-β1 upregulation in lung fibrotic changes and lung stiffening during the process of HCC lung metastases.

Recent findings have demonstrated that CEMIP exhibits pro-fibrotic functions and acts as a therapeutic target of pirfenidone for the treatment of mild-to-moderate idiopathic pulmonary fibrosis (IPF) [34]. Next, we utilized the Auto Dock docking tool to illustrate the potential binding relationships between pirfenidone and target CEMIP. According to molecular docking predictions (Supplementary Fig. S3B), CEMIP was a reliable target protein of pirfenidone. Pirfenidone has been reported to inhibit the TGF-β signaling pathway [34–36], which contributes to tissue fibrosis [37]. Therefore, we hypothesized that pirfenidone regulated CEMIP expression via the TGF-β signaling pathway during the process of HCC lung metastasis.

In support of our hypothesis, immunofluorescence staining results showed that CEMIP could enhance TGF-β1-induced Smad2 nuclear translocation in MRC5 cells, while pirfenidone could effectively impair TGF-β1-induced Smad2/3 nuclear accumulation, although its inhibition was not as significant as that of the TGF-β1 inhibitor (Supplementary Fig. S3C and Fig. 5C). We also observed that treatment with pirfenidone may reduce the total cellular levels of Smad2 and Smad3 proteins (Fig. 5D). Moreover, CEMIP silencing could mimic the inhibitory effects of pirfenidone (Fig. 5E). Collectively, we proved that pirfenidone blocks TGF-β1-induced fibrotic changes via the inhibition of the canonical Smad2/3 signaling pathway as well as the reduction in CEMIP expression levels, thus ameliorating the tumor mechano-environment.

**Fig. 6:**
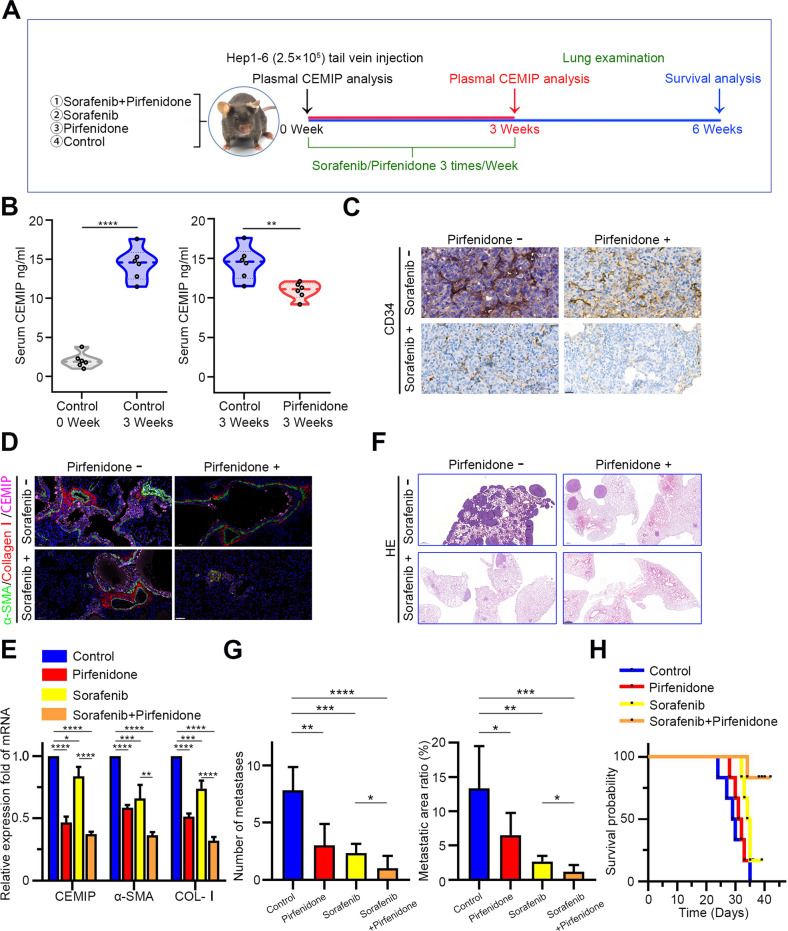
The combination of pirfenidone and sorafenib suppresses metastatic angiogenesis and prolongs survival. **A** Schematic of the animal experiments conducted using C57BL/6J mice. **B** Left**:** Plasma CEMIP levels of mice exhibiting lung metastasis and control mice. Right**:** Plasma CEMIP levels of pirfenidone-naive and pirfenidone-treated mice. **C** CD34 expression in paraffin sections of metastasized foci. Scale bar = 20 μm. **D** Immunostaining of COL-I (red), α-SMA (green), and CEMIP (pink) on lung tissues adjacent to the metastasized foci. Scale bar = 50 μm. **E** The expression of stiffness-associated markers expressed in lung sections obtained from 4-week-old mice in different treatment groups was determined by qRT-PCR analysis. **F**, **G** Comparison of metastatic colony number and area in the lungs of mice at week 4. Scale bar = 500 μm. **H** Kaplan–Meier curves of cumulative overall survival in mice receiving different therapies.

Currently, anti-angiogenic therapies have shown limited efficacy in the management of HCC lung metastases. Based on our in vitro findings, we further evaluated whether the anti-fibrotic effects of pirfenidone could augment the efficacy of sorafenib in vivo. We established a lung metastasis model and measured the vascular density in lung metastases in vivo (Fig. 6A). As shown in Fig. 6B, plasma CEMIP expression levels were significantly upregulated as the tumor volume increased, while pirfenidone treatment could significantly downregulate the circulating CEMIP level. As expected, sorafenib exhibited a robust inhibition of angiogenesis as evidenced by reduced vascular density, while the inhibitory effect was further enhanced by pirfenidone, although the effect of pirfenidone alone was mild (Fig. 6C). These in vivo findings are consistent with the in vitro findings that pirfenidone strengthened the inhibition of tube formation by sorafenib in HUVECs (Fig. 4F). Furthermore, pirfenidone treatment alone reduced metastasis stiffness (assessed by measuring COL-I, aSMA, and CEMIP levels in the same therapy group), and this effect was further consolidated in combination with sorafenib (Fig. 6D, E). Consistently, metastatic colony numbers and areas were significantly decreased in the combination therapy group compared with the sorafenib and pirfenidone monotherapy (Fig. 6F, G). These findings were supported by further survival analysis that the combination of sorafenib and pirfenidone demonstrated the greatest survival benefit (Fig. 6H). Taken together, our results suggest that pirfenidone not only strengthens the anti-angiogenesis effect of sorafenib but also reduces lung parenchymal stiffening, resulting in significantly enhanced survival benefits in combination with sorafenib.

### CEMIP levels are upregulated in HCC specimens and may indicate a lung fibrotic change in HCC patients with lung metastasis

**Fig. 7:**
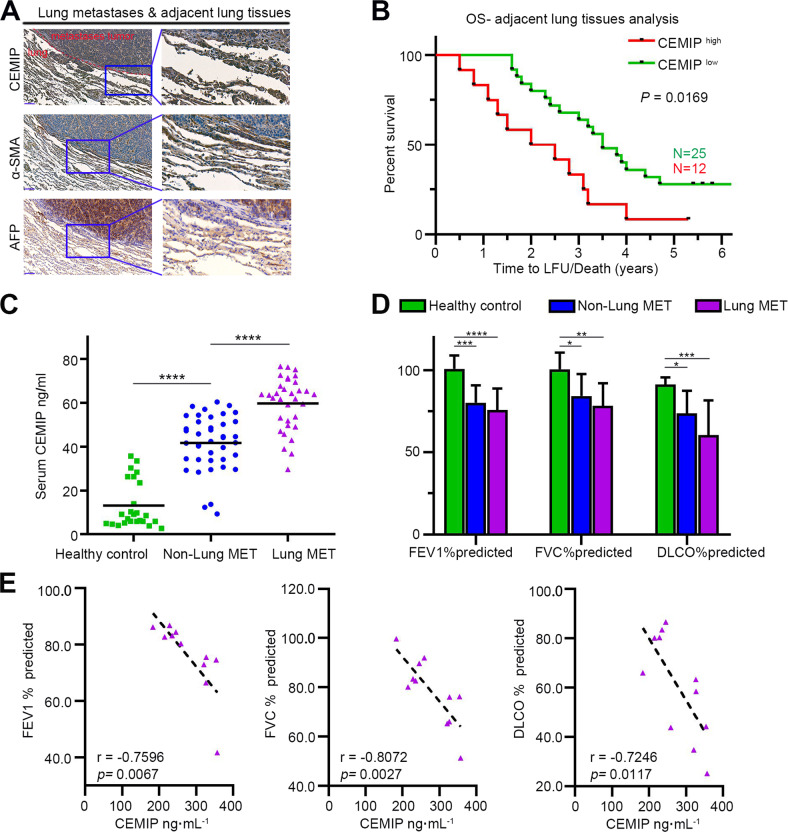
CEMIP expression is correlated with HCC lung metastasis in clinical specimens. **A** IHC staining of CEMIP, HCC markers (AFP), and fibroblast markers (α-SMA) on serial sections of human HCC lung metastatic tissue samples. Scale bar, 100 μm. **B** Kaplan–Meier survival curve for lung metastasis patients depicting time to last follow-up (LFU) or death from the time of primary tumor diagnosis based on low (green) or high (red) CEMIP expression levels in adjacent lung tissues. **C** CEMIP levels in the plasma obtained from healthy donors and primary HCC patients with or without lung metastasis. **D** Pulmonary function parameters in healthy donors and primary HCC patients with or without lung metastasis (FEV1, forced expiratory volume in 1 s; FVC, forced vital capacity; DLCO, diffusing capacity of carbon monoxide). **E** Relationship between plasma CEMIP levels and pulmonary function parameters in HCC patients with lung metastasis.

To further confirm the effect of CEMIP expression in lung metastatic tumors (MTs), we performed immunohistochemistry (IHC) triple-staining of CEMIP and fibroblast marker (α-SMA) analysis on serial sections of human lung metastatic tissues obtained from HCC patients. As shown in Fig. 7A, CEMIP immunoreactivity was mainly observed in tumor cells, alveolar type II cells, and some fibroblasts around the tumor. Moreover, high expression of CEMIP in adjacent lung tissues resulted in a poorer prognosis for HCC patients with lung metastasis (*P* = 0.0169) (Fig. 7B).

Next, we investigated CEMIP expression in different serum samples (25 healthy controls, 40 HCC patients without lung metastasis, and 33 HCC patients with lung metastasis). Compared to healthy controls, circulating levels of CEMIP were significantly upregulated in patients with HCC, and the levels were further elevated in patients with lung metastasis (Fig. 7C).

Interstitial fibrotic changes in the lung are demonstrated by decreased pulmonary compliance. Therefore, we measured pulmonary function parameters in HCC patients and healthy controls. As shown in Fig. 7D and Supplementary File 1: Supplementary Table S1, compared to healthy controls, HCC patients showed a typical restrictive ventilatory abnormality and decline in lung function as demonstrated by decreasing FEV1, FVC%, and DLCO%. Next, the relationships between serum CEMIP level and lung function parameters were analyzed. For patients without lung metastasis, we observed a negative correlation with predicted DLCO% (Supplementary Fig. S3D). For patients with lung metastasis, the serum CEMIP level showed a significant negative correlation with predicted FEV1%, FVC%, and DLCO% (Fig. 7E). Taken together, these data show that upregulated CEMIP is indicative of the severity of interstitial lung changes associated with HCC lung metastasis and may serve as a potential metastatic biomarker and therapeutic target for HCC.

## Discussion

It is important to obtain an insight into the stiffness of the microenvironment of lung metastases, which would provide us opportunities for the early diagnosis and therapeutic targeting of HCC lung metastasis. In this study, we demonstrated for the first time that matrix rigidity and ECM-related alterations in the lung microenvironment might generate a metastatic niche that supports HCC colonization. We characterized CEMIP, a pro-metastatic protein enriched in primary tumors in the liver that might activate LFs and therefore increase tumorigenic ECM stiffness in the lungs. In addition, we demonstrated that CEMIP-mediated ECM stiffening could promote tumor progression in a stiffness-dependent manner. Encouragingly, the anti-fibrotic drug pirfenidone, approved by the FDA for the treatment of IPF, may effectively decrease CEMIP expression and alleviate ECM stiffness in lung metastases. Therefore, our findings uncovered the mechanism underlying the formation of the pro-metastatic niche and provided a new ECM-targeted therapeutic strategy for the treatment of lung metastasis in HCC patients.

The expression of CEMIP has previously been associated with tumor progression [38, 39] and fibrotic diseases [40], our study reveals a previously unrecognized role of CEMIP-mediated ECM stiffness in lung metastasis in HCC. Though the precise underlying mechanism needs to be investigated further, we observed that, as a secreted protein, CEMIP was detectable in the serum of HCC patients and exerts functions via paracrine mechanisms. Notably, tumor cells could release exosomal CEMIP to promote metastasis [16]. Our work encourages further investigation into the role of exosomal CEMIP in metastatic HCC, which makes our findings clinically relevant and supports the potential application of CEMIP as a prognostic marker of lung metastasis in HCC patients.

Because the role of ECM stiffening in cancer progression has become increasingly clearer, the mechanotherapeutics of solid tumors have attracted increased attention [41, 42]. A stiff environment might precede tumor seeding and metastasis [43]. Hence, targeted therapies directed against the establishment of pre-metastatic niches and ECM remodeling events could potentially prevent and eliminate cancer metastasis. In recent years, several preclinical and clinical studies have explored the efficacy of using the mechano-therapeutic approach in cancer; promising outcomes, including those of drugs targeting CTGF, TGF-β signaling, and LOX-mediated matrix stiffening, have been achieved [44]. Notably, pirfenidone, the first-line medication for IPF [45], has been demonstrated to suppress TGF-β signaling and was proven to have antitumor effects in pancreatic cancer [46] and lung metastasis in triple-negative breast cancer [47]. Thus, even though we focused on metastatic HCC, similar mechanisms could play a role in other tumor entities. Recent findings have shown that CEMIP could influence the stromal environment by participating in the catabolism of ECM components [46], and was identified as a target of pirfenidone [48]. Our in vivo experimental data demonstrate that pirfenidone treatment significantly reduced ECM stiffness at the lung metastasis site. However, ECM-targeted therapy alone is insufficient to effectively overcome tumor metastasis. In this context, an antitumor strategy involving a combination of drugs is emerging as a better treatment option. Since it has previously been demonstrated that the stiffening of the tumor ECM promotes tumor angiogenesis [49] and drug resistance [50]. Therefore, it is important to maximize the potential of anti-angiogenic therapy, as it would emphasize the importance of a tumor microenvironment-specific therapeutic strategy. Sorafenib, a multi-kinase inhibitor of VEGF/VEGFR and RAF/MEK/ERK pathways that exhibits anti-angiogenic and anti-proliferative activities, is recommended as a standard first-line drug for patients with advanced-stage HCC. We observed that the co-administration of pirfenidone and sorafenib resulted in a combined effect in vivo, leading to the maximal suppression of vascular density and significantly decreased HCC lung metastasis, compared to that observed upon treatment with sorafenib alone. Histologically, combination therapy resulted in a considerable reduction in collagen I synthesis, α-SMA positive areas, and CEMIP expression levels. These findings suggest that the effects of pirfenidone in vivo include suppressed fibroblast proliferation and stromal component production. Besides, we demonstrated that CEMIP could enhance the extent of tumor metastasis and angiogenic potential. Therefore, the antitumor effects of pirfenidone are partly mediated by the inhibition of CEMIP production. Notably, no significant change in the vital signs (including body weight, fur condition, behaviors, food intake, and fecal condition) of mice was observed during the combination treatment. In addition, to assess the safety for the liver and kidney, the blood biochemical indexes of C57 mice in different groups were also measured at 4 weeks. Alanine transaminase (ALT), aspartate transaminase (AST), total bilirubin (TBILI), blood urea nitrogen (BUN), and creatinine (CREA) did not show an obvious difference in all groups of mice, indicating that this combination therapy was unlikely to cause damages to the liver and kidney (Supplementary Table S4). For the first time, we found that the co-administration of sorafenib, which inhibits tumor growth and angiogenesis, and pirfenidone, which targets extracellular matrix stiffness, had synergistic antitumor effects. Therefore, we provide a novel strategy for identifying and overcoming stiffness-mediated resistance mechanisms during lung metastasis in HCC patients.

In conclusion, the present study identified the role of CEMIP in metastatic HCC. Our findings suggest that CEMIP is a promising biomarker and therapeutic target for HCC lung metastasis. Pirfenidone may reduce extracellular matrix stiffness and enhance the efficacy of sorafenib treatment. Therefore, this study elucidates a new molecular mechanism underlying the crosstalk between tumor cells and the mechanical environment that promotes lung metastasis and offers an alternative option for the treatment of lung metastasis in HCC patients using combination therapy.

## Supplementary information

supplementary information

Supplementary file 2

Supplementary Figure 1

Supplementary Figure 2

Supplementary Figure 3

Original Data File

checklist

## Data Availability

All datasets generated and analyzed during this study are included in this published article and its Supplementary Information files. Additional data are available from the corresponding author upon reasonable request.

## References

[1] Sung H, Ferlay J, Siegel RL, Laversanne M, Soerjomataram I, Jemal A (2021). Global cancer statistics 2020: GLOBOCAN estimates of incidence and mortality worldwide for 36 cancers in 185 countries. CA Cancer J Clin.

[2] Nakano M, Tanaka M, Kuromatsu R, Nagamatsu H, Tajiri N, Satani M (2015). Sorafenib for the treatment of advanced hepatocellular carcinoma with extrahepatic metastasis: a prospective multicenter cohort study. Cancer Med.

[3] Finn RS, Qin S, Ikeda M, Galle PR, Ducreux M, Kim TY (2020). Atezolizumab plus bevacizumab in unresectable hepatocellular carcinoma. N Engl J Med.

[4] Peinado H, Zhang H, Matei IR, Costa-Silva B, Hoshino A, Rodrigues G (2017). Pre-metastatic niches: organ-specific homes for metastases. Nat Rev Cancer.

[5] Theocharis AD, Skandalis SS, Gialeli C, Karamanos NK (2016). Extracellular matrix structure. Adv Drug Deliv Rev.

[6] Sinha S, Cao Y, Dutta S, Wang E, Mukhopadhyay D (2010). VEGF neutralizing antibody increases the therapeutic efficacy of vinorelbine for renal cell carcinoma. J Cell Mol Med.

[7] Joyce MH, Lu C, James ER, Hegab R, Allen SC, Suggs LJ (2018). Phenotypic basis for matrix stiffness-dependent chemoresistance of breast cancer cells to doxorubicin. Front Oncol.

[8] Egeblad M, Rasch MG, Weaver VM (2010). Dynamic interplay between the collagen scaffold and tumor evolution. Curr Opin Cell Biol.

[9] Shen Y, Wang X, Lu J, Salfenmoser M, Wirsik NM, Schleussner N (2020). Reduction of liver metastasis stiffness improves response to bevacizumab in metastatic colorectal cancer. Cancer Cell.

[10] Cortes E, Lachowski D, Robinson B, Sarper M, Teppo JS, Thorpe SD, et al. Tamoxifen mechanically reprograms the tumor microenvironment via HIF-1A and reduces cancer cell survival. EMBO Rep. 2019;20:e46557. 10.15252/embr.201846557 PMC6322388 30538116

[11] Picozzi V, Alseidi A, Winter J, Pishvaian M, Mody K, Glaspy J, et al. Gemcitabine/nab-paclitaxel with pamrevlumab: a novel drug combination and trial design for the treatment of locally advanced pancreatic cancer. ESMO Open. 2020;5:e000668. 10.1136/esmoopen-2019-000668 PMC7440698 32817130

[12] Liu J, Yan W, Han P, Tian D (2021). The emerging role of KIAA1199 in cancer development and therapy. Biomed Pharmacother.

[13] Yoshida H, Nagaoka A, Nakamura S, Tobiishi M, Sugiyama Y, Inoue S (2014). N-terminal signal sequence is required for cellular trafficking and hyaluronan-depolymerization of KIAA1199. FEBS Lett.

[14] Abe S, Usami S, Nakamura Y (2003). Mutations in the gene encoding KIAA1199 protein, an inner-ear protein expressed in Deiters’ cells and the fibrocytes, as the cause of nonsyndromic hearing loss. J Hum Genet.

[15] Zhang P, Song Y, Sun Y, Li X, Chen L, Yang L (2018). AMPK/GSK3beta/beta-catenin cascade-triggered overexpression of CEMIP promotes migration and invasion in anoikis-resistant prostate cancer cells by enhancing metabolic reprogramming. FASEB J.

[16] Matsuzaki S, Tanaka F, Mimori K, Tahara K, Inoue H, Mori M (2009). Clinicopathologic significance of KIAA1199 overexpression in human gastric cancer. Ann Surg Oncol.

[17] Rodrigues G, Hoshino A, Kenific CM, Matei IR, Steiner L, Freitas D (2019). Tumour exosomal CEMIP protein promotes cancer cell colonization in brain metastasis. Nat Cell Biol.

[18] Xu Y, Xu H, Li M, Wu H, Guo Y, Chen J (2019). KIAA1199 promotes sorafenib tolerance and the metastasis of hepatocellular carcinoma by activating the EGF/EGFR-dependent epithelial-mesenchymal transition program. Cancer Lett.

[19] Kwapiszewska G, Gungl A, Wilhelm J, Marsh LM, Thekkekara Puthenparampil H, Sinn K, et al. Transcriptome profiling reveals the complexity of pirfenidone effects in idiopathic pulmonary fibrosis. Eur Respir J. 2018;52:1800564. 10.1183/13993003.00564-2018 30166321

[20] Asano S, Ito S, Takahashi K, Furuya K, Kondo M, Sokabe M, et al. Matrix stiffness regulates migration of human lung fibroblasts. Physiol Rep. 2017;5:e13281. 10.14814/phy2.13281 PMC5430127 28507166

[21] Kong J, Tian H, Zhang F, Zhang Z, Li J, Liu X (2019). Extracellular vesicles of carcinoma-associated fibroblasts creates a pre-metastatic niche in the lung through activating fibroblasts. Mol Cancer.

[22] Soikkeli J, Podlasz P, Yin M, Nummela P, Jahkola T, Virolainen S (2010). Metastatic outgrowth encompasses COL-I, FN1, and POSTN up-regulation and assembly to fibrillar networks regulating cell adhesion, migration, and growth. Am J Pathol.

[23] Liu Y, Cao X (2016). Characteristics and significance of the pre-metastatic niche. Cancer Cell.

[24] Kaplan RN, Riba RD, Zacharoulis S, Bramley AH, Vincent L, Costa C (2005). VEGFR1-positive haematopoietic bone marrow progenitors initiate the pre-metastatic niche. Nature..

[25] Meads MB, Gatenby RA, Dalton WS (2009). Environment-mediated drug resistance: a major contributor to minimal residual disease. Nat Rev Cancer.

[26] Kalluri R, Zeisberg M (2006). Fibroblasts in cancer. Nat Rev Cancer.

[27] Bordeleau F, Mason BN, Lollis EM, Mazzola M, Zanotelli MR, Somasegar S (2017). Matrix stiffening promotes a tumor vasculature phenotype. Proc Natl Acad Sci USA.

[28] Booth AJ, Hadley R, Cornett AM, Dreffs AA, Matthes SA, Tsui JL (2012). Acellular normal and fibrotic human lung matrices as a culture system for in vitro investigation. Am J Respir Crit Care Med.

[29] Hinz B (2012). Mechanical aspects of lung fibrosis: a spotlight on the myofibroblast. Proc Am Thorac Soc.

[30] Yao Y, Hu C, Song Q, Li Y, Da X, Yu Y (2020). ADAMTS16 activates latent TGF-beta, accentuating fibrosis and dysfunction of the pressure-overloaded heart. Cardiovasc Res.

[31] Shi Y, Massague J (2003). Mechanisms of TGF-beta signaling from cell membrane to the nucleus. Cell..

[32] Phillips PA, McCarroll JA, Park S, Wu MJ, Pirola R, Korsten M (2003). Rat pancreatic stellate cells secrete matrix metalloproteinases: implications for extracellular matrix turnover. Gut..

[33] Attisano L, Wrana JL (2002). Signal transduction by the TGF-beta superfamily. Science..

[34] Oku H, Shimizu T, Kawabata T, Nagira M, Hikita I, Ueyama A (2008). Antifibrotic action of pirfenidone and prednisolone: different effects on pulmonary cytokines and growth factors in bleomycin-induced murine pulmonary fibrosis. Eur J Pharm.

[35] Liu H, Drew P, Gaugler AC, Cheng Y, Visner GA (2005). Pirfenidone inhibits lung allograft fibrosis through L-arginine-arginase pathway. Am J Transpl.

[36] Dosanjh A, Ikonen T, Wan B, Morris RE (2002). Pirfenidone: A novel anti-fibrotic agent and progressive chronic allograft rejection. Pulm Pharm Ther.

[37] Akhurst RJ, Hata A (2012). Targeting the TGFbeta signalling pathway in disease. Nat Rev Drug Discov.

[38] Shen F, Zong ZH, Liu Y, Chen S, Sheng XJ, Zhao Y (2019). CEMIP promotes ovarian cancer development and progression via the PI3K/AKT signaling pathway. Biomed Pharmacother.

[39] Li L, Yan LH, Manoj S, Li Y, Lu L (2017). Central role of CEMIP in tumorigenesis and its potential as therapeutic target. J Cancer.

[40] Deroyer C, Charlier E, Neuville S, Malaise O, Gillet P, Kurth W (2019). CEMIP (KIAA1199) induces a fibrosis-like process in osteoarthritic chondrocytes. Cell Death Dis.

[41] Elez E, Kocakova I, Hohler T, Martens UM, Bokemeyer C, Van Cutsem E (2015). Abituzumab combined with cetuximab plus irinotecan versus cetuximab plus irinotecan alone for patients with KRAS wild-type metastatic colorectal cancer: the randomised phase I/II POSEIDON trial. Ann Oncol.

[42] Sheridan C (2019). Pancreatic cancer provides testbed for first mechanotherapeutics. Nat Biotechnol.

[43] Costa-Silva B, Aiello NM, Ocean AJ, Singh S, Zhang H, Thakur BK (2015). Pancreatic cancer exosomes initiate pre-metastatic niche formation in the liver. Nat Cell Biol.

[44] Lampi MC, Reinhart-King CA. Targeting extracellular matrix stiffness to attenuate disease: from molecular mechanisms to clinical trials. Sci Transl Med. 2018;10:eaao0475. 10.1126/scitranslmed.aao0475 29298864

[45] Selvaggio AS, Noble PW (2016). Pirfenidone initiates a new era in the treatment of idiopathic pulmonary fibrosis. Annu Rev Med.

[46] Kozono S, Ohuchida K, Eguchi D, Ikenaga N, Fujiwara K, Cui L (2013). Pirfenidone inhibits pancreatic cancer desmoplasia by regulating stellate cells. Cancer Res.

[47] Takai K, Le A, Weaver VM, Werb Z (2016). Targeting the cancer-associated fibroblasts as a treatment in triple-negative breast cancer. Oncotarget..

[48] Maher TM, Corte TJ, Fischer A, Kreuter M, Lederer DJ, Molina-Molina M (2020). Pirfenidone in patients with unclassifiable progressive fibrosing interstitial lung disease: a double-blind, randomised, placebo-controlled, phase 2 trial. Lancet Respir Med.

[49] Jain RK, Martin JD, Stylianopoulos T (2014). The role of mechanical forces in tumor growth and therapy. Annu Rev Biomed Eng.

[50] Schrader J, Gordon-Walker TT, Aucott RL, van Deemter M, Quaas A, Walsh S (2011). Matrix stiffness modulates proliferation, chemotherapeutic response, and dormancy in hepatocellular carcinoma cells. Hepatology..

