# Supplementary material for: Inhibition of CEMIP potentiates the effect of sorafenib on metastatic hepatocellular carcinoma by reducing the stiffness of lung metastases
Source: Cell Death Dis. 2023 Jan 13;14(1):25. doi: 10.1038/s41419-023-05550-4 (PMC9839779; doi:10.1038/s41419-023-05550-4)
Supplement: Supplementary file 1 — supplementary information [file 41419_2023_5550_MOESM1_ESM.docx]

**Supplementary file 1**

| **Supplementary Materials and Methods** |
| --- |
| **Supplementary Table S1.** Analysis of pulmonary function parameters in HCC patients and healthy controls |
| **Supplementary Table S2.** Antibodies. |
| **Supplementary Table S3.** Forward and reverse primers for RT-PCR. |
| **Supplementary Table S4.** Plasma biochemistry parameters in different treatment groups of tumor-bearing mice. |
| **Supplementary Figure legends** |

**Supplementary Materials and Methods**

**Specimens and Cell lines**

Tissue and serum samples were obtained from patients in the Second Affiliated Hospital of Guangzhou Medical University. Serum specimens collected from healthy donors were obtained from the physical examination center. All procedures were conducted with the approval of the Ethical Committee of Second Affiliated Hospital of Guangzhou Medical University. Patient consent was obtained before the start of the study. The privacy rights of human subjects are always observed.Investigators were blinded to the group allocation during the experiment and when assessing the outcome.

The human liver cancer cell lines Hep3B, LM3, Huh7 cells, and the mouse liver cancer cell line Hepa1-6 were purchased from the National Collection of Authenticated Cell Cultures and cultured in dulbecco's modified eagle medium (DMEM). The human fetal lung fibroblast cell line MRC5 was purchased from Sciencell Company and cultured in Minimum Essential Medium Eagle (MEM). The culture media were supplemented with 10% fetal bovine serum and 1% penicillin/streptomycin. Cells were maintained in a 5% CO2 and 95% air incubator.

**Animal Studies**

Pre-metastatic model: using BALB/c nude mice aged 4 weeks old (about 18 g, male, Guangdong Medical Laboratory Animal Center). Mice were randomly divided into groups. 1× 10^6^ Hep3B cells were injected under the capsule of the left hepatic lobe of male BALB/c nude mice. Mice were divided into three groups according to the types of transplanted tumor cells: the CEMIP-overexpressed group (Hep3B cells infected with a CEMIP overexpression lentivirus), LV-control group (cells were transfected with negative control lentivirus), and blank control group (untreated cells), with five mice in each group. The nude mice were raised in sterile conditions, then euthanized. Three sets of experiments were conducted. In the first set, the nude mice were raised in sterile conditions for 3 weeks after orthotopic xenografts were performed. In the second set, the nude mice were raised in sterile conditions for 5 weeks after orthotopic xenografts were performed. In the third set of experiments, mice were raised until they died naturally and survival was analyzed. The lung tissues were removed and fixed with 4% paraformaldehyde solution overnight, then embedded into OCT. The whole blood of each mouse was collected for plasma Elisa experiments.

Lung metastasis model: Hep1-6 cells were injected into the tail vein of C57BL/6 J mice aged 5 weeks old (about 18g, male, Guangdong Medical Laboratory Animal Center). Mice were divided into four groups based on the medications administered: Sorafenib group (30mg/kg/time), Pirfenidone group (125mg/kg/time), Sorafenib+Pirfenidone combination group, control group, with six mice in each group. Drugs were used three times a week for three weeks. Mice were raised until they died naturally and the lungs were fixed and embedded into OCT. Microvessel density (MVD) of metastasis in the lung tissues was evaluated by counting CD34-labeled vessels. Investigators were blinded to the group allocation during the experiment and when assessing the outcome. All animal studies were carried out under the research protocol A-2020-037, approved by the Animal Care and Use Committee at the Second

Affiliated Hospital of Guangzhou Medical University.

**Histology, Immunohistochemistry and Immunofluorescence**

Paraffin-embedded tissue samples were cut into 4 µm-thick sections and stained with hematoxylin eosin (H&E), Sirius red, Masson’s trichrome and immunohistochemistry according to standard procedures. For dual immunofluorescence staining, sections or cells fixed with ice-cold methanol were co-stained with primary antibodies and appropriate secondary antibodies labeled with either Alexa Fluor 488 or Alexa Fluor 555, according to the manufacturer’s instructions. Nuclei were stained with DAPI. Primary antibodies are listed in the table below. The slices were observed using a Zeiss LM800 confocal microscope.

**Shear wave elastography**

To perform lung elastography, the animals were put in a supine position. Lung stiffness measurements were obtained using the ACUSON S2000 ultrasound system (Siemens Medical Solutions, Mountain View, CA, USA) with a 4-9 MHz linear transducer. A trained radiologist with 3 years of experience conducted all SWE examinations. SWE was performed with an elasticity range of 0 to 6 m/s. The elastic sampling boxes contained unilateral lung tissue at least, and the measurement sampling boxes were uniform which was the default of the machine. Without any compression on the animal surface, the elastic sampling boxes were maintained for at least 3s to generate stable elastic images. All shear wave velocity (SWV) measurements were recorded. Notably, the SWV values represented the averages of three measurements of the measurement sampling box at each position.

**Migration assay (*in vitro*)**

Cell migration was evaluated using 24-well chambers(Corning Inc., Corning, MA, USA). Cells were seeded at a density of 5×104 into the upper chamber in 200μl DMEM medium without FBS, and 600μl complete DMEM medium was added to the bottom chamber. After incubation for 24hr, the migratory cells were fixed and stained with 0.1% crystal violet (C0121, Beyotime).

**Detection of Cell Proliferation**

Ethynyl-2-deoxyuridine (EdU) cell proliferation detection kits (RiboBio Co., Ltd., Guangzhou, Guangdong, China) were applied for cell proliferation analysis. In brief, cells were plated in 96-well plates with 2 × 10^4^ cells/well for 24 h, and then added with 50 µM EdU at 37°C. After 2 h, cells were fixed for 15 min in 4% paraformaldehyde and permeabilized for 20 min using 0.5% Triton X-100. After PBS washing, cells were treated with ApolloR reaction cocktail (100 μL) for 30 min and added with Hoechst for nuclei counterstaining for 30 min. Finally, cells were imaged under a microscope (Olympus, Tokyo, Japan).

**Capillary-like tubule structure formation assay (*in vitro*)**

In vitro angiogenesis was assessed by Matrigel capillary-like tubule structure formation assay. Matrigel (corning 356234, USA) or polyacrylamide hydrogels was pipetted into pre-chilled 96-well plates (50 μL matrigel per well) and polymerized for 40 min at 37°C. HUVECs were seeded onto the solidified gel, and the endothelial tubes were counted under photomicroscope. Capillary tubule branch points were counted in six randomly selected fields per well.

**Western blotting**

In brief, proteins were separated by SDS-PAGE gel and transferred onto a polyvinylidene fluoride microporous membrane (PVDF, 0036, Millipore). The membranes were probed with primary antibody (see Table 2 below) overnight at 4°C and Horseradish Peroxidase conjugated secondary antibody for 2 hr at room temperature. A chemiluminescence kit (102031714, 102031696, Bio-Rad) was added to the membranes and the blots were visualized using a ChemiDoc (6000Touch, Clinx Science Instruments Co., Ltd).

**ELISA**

The level of CEMIP in serums sample and cell supernatant sample was measured using ELISA kit (SY-H07950/SY-M05203, SHUANGYING BIOLOGICAL) according to the instructions of the manufacturer. The concentration of each sample in well was determined by interpolation from a standard curve.

**Quantitative real-time PCR**

Total RNA was isolated using the TransZol Up Plus RNA Kit (ER501-01, TransGen Biotech). cDNA synthesis was performed using All-in-One First-Strand cDNA Synthesis SuperMix (AT341-02, TransGen Biotech). qRT-PCR was prepared using PerfectStart Green qPCR SuperMix (AQ601-02, TransGen Biotech) used for gene specifific amplifification and run on a LightCycler480 II detection system (31756, Roche). The sequences of primers for RT-PCR are listed in Table S3.

**Lentiviral vectors and cell transduction**

The interference sequences targeting CEMIP (cgAATGAAGATCATCAAGAAT) and CEMIP overexpression sequence (see Supplementary file 2) were designed and purchased from the Shanghai GeneChem, Co. Ltd., China. Cells were transduction with lentiviral vector according to the manufacturer’s protocol.

**Supplementary Table S1**. Analysis of predicted FEV1%, predicted FVC%, and predicted DLCO% in HCC patients and healthy controls (X ± s) using one-way ANOVA.

| Study population | n | Predicted FEV1% | Predicted FVC% | Predicted DLCO% |
| --- | --- | --- | --- | --- |
| Healthy controls | 10 | 100.82±7.80 | 100.64±9.62 | 91.38±4.10 |
| HCC non-lung metastasis patients | 10 | 80.23±10.07 | 84.4±12.69 | 73.8±13.03 |
| HCC lung metastasis patients | 11 | 75.85±12.40 | 78.41±13.07 | 60.54±20.20 |
| F |  | 15.4 | 8.672 | 11.01 |
| p-value |  | ＜0.0001 | 0.0012 | 0.0003 |

**Supplementary Table S2.** Antibodies.

| Reagent | Company | Catalog |
| --- | --- | --- |
| anti-CEMIP | Proteintech | 21129-1-AP |
| anti-α-SMA | Proteintech | 55135-1-AP |
| anti-Fibronectin | Wanleibio | WL00712a |
| anti-Collagen Ⅰ | Proteintech | 14695-1-AP |
| anti-LOX | Proteintech | 17958-1-AP |
| anti-MMP9 | Proteintech | 10375-2-AP |
| anti-MMP7 | Proteintech | 10374-2-AP |
| anti-TGF-β1 | Abcam | Ab215715 |
| anti-Periostin | Proteintech | 66491-1-Ig |
| anti-VEGFR 1 | GeneTex | GTX01186 |
| anti-CD34 | Abcam | Ab8158 |
| anti-Smad2/3 | CST | #8685 |
| Sorafenib | MedChem Express | HY-10201S1 |
| Pirfenidone | MedChem Express | [HY-B0673](https://www.medchemexpress.cn/Pirfenidone.html) |
| CEMIP Elisa kit | SHUANGYING BIOLOGICAL | SY-H07950/SY-M05203 |
| TGF-β1 ELISA kit | NeoBioscience | EHC107b |

**Supplementary Table S3.** Forward and reverse primers for RT-PCR.

| **Primers name** |  | **Sequence (5’-3’)** |
| --- | --- | --- |
| m-CEMIP | Forward | TCTGTCGCAGTGAATGATGAAGGC |
|  | Reverse | GCTCCAAGGGTGTCTAAATCCAAGG |
| m-Fibronectin | Forward | CTATAGGATTGGAGACACGTGG |
|  | Reverse | CTGAAGCACTTTGTAGAGCATG |
| m-LOX | Forward | CTACGATTTCCGCAAAGAGTGAAGAAC |
|  | Reverse | TGTTGGCATCAAGCAGGTCATAGTG |
| m-MMP9 | Forward | CAAAGACCTGAAAACCTCCAAC |
|  | Reverse | GACTGCTTCTCTCCCATCATC |
| m-α-SMA | Forward | AATGACCCAGATTATGTTTGAGACCT |
|  | Reverse | TCCAGAGTCCAGCACAATACCAG |
| m-Collagen Ⅰ | Forward | GACAGGCGAACAAGGTGACAGAG |
|  | Reverse | CAGGAGAACCAGGAGAACCAGGAG |
| h-FAP | Forward | TGTTCCAGCAATGATAGCCTCAAGTG |
|  | Reverse | TCCCATGTCTGCCAGTCTTCCC |
| h-TGF-β1 | Forward | TCCTGGCGATACCTCAGCAA |
|  | Reverse | CAATTTCCCCTCCACGGCTC |
| h-α-SMA | Forward | GTGTTGCCCCTGAAGAGCAT |
|  | Reverse | GCTGGGACATTGAAAGTCTCA |
| h-Collagen Ⅰ | Forward | GAGGGCCAAGACGAAGACATC |
|  | Reverse | CAGATCACGTCATCGCACAAC |

**Supplementary Table S4.** Serum biochemistry parameters in different treatment groups of tumor-bearing mice (Dunnett’s test, *P*＞0.05).

|  | Control | pirfenidone | sorafenib | pirfenidone+sorafenib |
| --- | --- | --- | --- | --- |
| AST (U/L) | 122.56±5.87 | 120.33±3.27 | 119.53±8.80 | 120.06±7.20 |
| ALT (U/L) | 104.9±5.99 | 100.16±6.18 | 100.73±8.25 | 99.83±9.07 |
| TBILI (μmol/L) | 23.16±1.17 | 22.3±1.9 | 20.54±1.50 | 21.9±0.26 |
| CREA (μmol/L) | 87.06±3.55 | 79.7±2.96 | 84.9±3.55 | 86.03±4.40 |
| BUN (mmol/L) | 5.23±0.12 | 5.30±0.22 | 5.07±0.11 | 5.45±0.38 |

**Supplementary Figure 1. CEMIP promotes tumor progression in a stiffness-dependent manner**

**(A)** Representative immunohistochemistry images illustrating CEMIP expression for each scoring category in patient tumor samples. Samples exhibiting no (0) or low (1 and 2) levels of CEMIP staining were considered as CEMIP-low (green) samples. Samples displaying high expression levels (3 and 4) of CEMIP were considered as CEMIP-high (red) samples. Scale bars, 50 μm.

**(B)** Quantification of VEGFR1 levels of the nude mice lung tissue by calculating relative fluorescence intensity.

**(C)** Stiffness maps and stiffness distribution of lung tissues were measured using AFM. Representative Hematoxylin-Eosin staining stained images of lung metastasis at 5 weeks. Scale bar = 50 μm.

**(D)** Huh7 cells were cultured on polyacrylamide hydrogels with different levels of stiffness. Cell Counting Kit-8 (CCK8) solution was added, and absorbance at 450 nm was measured after 3 hours.

**(E)** The expression levels of CCND1 in Huh7 cells cultured on polyacrylamide hydrogels with different levels of stiffness were detected by qRT-PCR analysis.

**(F-H)** Effects of CEMIP-mediated substrate stiffness on malignant properties of cells: Cells were cultured on polyacrylamide hydrogels with different levels of stiffness for 72 h. Proliferation levels were evaluated using the EdU cell proliferation assay. The migratory ability of cancer cells was examined via the Transwell migration assay. Quantitative analysis of HUVEC tube formation. To extract some quantitative data, five independent fields were randomly selected and counted manually.

**Supplementary Figure 2. Clinical samples shows CEMIP mediated stiffening was associated with angiogenesis**

**(A)** The stiffness distribution in benign lung lesions and the microenvironment of HCC lung metastases is consistent with their histopathological results (collagen Ⅰ, α-SMA staining), and reveals nests of HCC cells that have evoked a stiff fibrous tissue response in lung metastases. High-resolution stiffness maps of lung tissues were constructed using AFM. The approximate stiffness mapping area was 60 × 60 μm. Scale bar = 50 μm.

**(B)** Representative images of stained CEMIP and CD34 in liver primary tumors, lung metastatic foci, and adjacent lung tissue samples (Case 1: low CEMIP expression levels; Case 2: medium CEMIP expression levels; Case 3: high CEMIP expression levels). Scale bar =50 μm.

**Supplementary Figure 3. TGF-β1/Smad signaling pathway may be involved in matrix stiffness-induced  fibrotic-like changes**

**(A)** ELISA (enzyme linked immunosorbent assay) was used to determine the levels of secreted TGF-β1 in supernatants of MRC5 cells with different levels of stiffness that were cultured on polyacrylamide hydrogels.

**(B)** The protein-ligand interaction between pirfenidone and CEMIP in the docking simulation (as it was a compound with a negative docking score (< -5), it was considered to exhibit binding activity).

**(C)** MRC5 fibroblasts were treated with 10 ng/mL TGF-β1 for 2 hours. Representative immunofluorescence images demonstrate the nuclear accumulation of Smad2 reduced after pirfenidone or TGF-β1 inhibitor treatment.

**(D)** Relationship between plasma CEMIP levels and pulmonary function parameters in HCC patients without lung metastasis.
