# Supplementary material for: Inhibition of CEMIP potentiates the effect of sorafenib on metastatic hepatocellular carcinoma by reducing the stiffness of lung metastases
Source: Cell Death Dis. 2023 Jan 13;14(1):25. doi: 10.1038/s41419-023-05550-4 (PMC9839779; doi:10.1038/s41419-023-05550-4)
Supplement: Supplementary file 2 [file 41419_2023_5550_MOESM2_ESM.docx]

**Supplementary file 2**

Human CEMIP overexpression sequence:

TTTGGCTTTTTTGTTAGACGAAGCTTGGGCTGCAGGTCGACTCTAGAGGATCCCGCCACCATGGGAGCTGCTGGGAGGCAGGACTTCCTCTTCAAGGCCATGCTGACCATCAGCTGGCTCACTCTGACCTGCTTCCCTGGGGCCACATCCACAGTGGCTGCTGGGTGCCCTGACCAGAGCCCTGAGTTGCAACCCTGGAACCCTGGCCATGACCAAGACCACCATGTGCATATCGGCCAGGGCAAGACACTGCTGCTCACCTCTTCTGCCACGGTCTATTCCATCCACATCTCAGAGGGAGGCAAGCTGGTCATTAAAGACCACGACGAGCCGATTGTTTTGCGAACCCGGCACATCCTGATTGACAACGGAGGAGAGCTGCATGCTGGGAGTGCCCTCTGCCCTTTCCAGGGCAATTTCACCATCATTTTGTATGGAAGGGCTGATGAAGGTATTCAGCCGGATCCTTACTATGGTCTGAAGTACATTGGGGTTGGTAAAGGAGGCGCTCTTGAGTTGCATGGACAGAAAAAGCTCTCCTGGACATTTCTGAACAAGACCCTTCACCCAGGTGGCATGGCAGAAGGAGGCTATTTTTTTGAAAGGAGCTGGGGCCACCGTGGAGTTATTGTTCATGTCATCGACCCCAAATCAGGCACAGTCATCCATTCTGACCGGTTTGACACCTATAGATCCAAGAAAGAGAGTGAACGTCTGGTCCAGTATTTGAACGCGGTGCCCGATGGCAGGATCCTTTCTGTTGCAGTGAATGATGAAGGTTCTCGAAATCTGGATGACATGGCCAGGAAGGCGATGACCAAATTGGGAAGCAAACACTTCCTGCACCTTGGATTTAGACACCCTTGGAGTTTTCTAACTGTGAAAGGAAATCCATCATCTTCAGTGGAAGACCATATTGAATATCATGGACATCGAGGCTCTGCTGCTGCCCGGGTATTCAAATTGTTCCAGACAGAGCATGGCGAATATTTCAATGTTTCTTTGTCCAGTGAGTGGGTTCAAGACGTGGAGTGGACGGAGTGGTTCGATCATGATAAAGTATCTCAGACTAAAGGTGGGGAGAAAATTTCAGACCTCTGGAAAGCTCACCCAGGAAAAATATGCAATCGTCCCATTGATATACAGGCCACTACAATGGATGGAGTTAACCTCAGCACCGAGGTTGTCTACAAAAAAGGCCAGGATTATAGGTTTGCTTGCTACGACCGGGGCAGAGCCTGCCGGAGCTACCGTGTACGGTTCCTCTGTGGGAAGCCTGTGAGGCCCAAACTCACAGTCACCATTGACACCAATGTGAACAGCACCATTCTGAACTTGGAGGATAATGTACAGTCATGGAAACCTGGAGATACCCTGGTCATTGCCAGTACTGATTACTCCATGTACCAGGCAGAAGAGTTCCAGGTGCTTCCCTGCAGATCCTGCGCCCCCAACCAGGTCAAAGTGGCAGGGAAACCAATGTACCTGCACATCGGGGAGGAGATAGACGGCGTGGACATGCGGGCGGAGGTTGGGCTTCTGAGCCGGAACATCATAGTGATGGGGGAGATGGAGGACAAATGCTACCCCTACAGAAACCACATCTGCAATTTCTTTGACTTCGATACCTTTGGGGGCCACATCAAGTTTGCTCTGGGATTTAAGGCAGCACACTTGGAGGGCACGGAGCTGAAGCATATGGGACAGCAGCTGGTGGGTCAGTACCCGATTCACTTCCACCTGGCCGGTGATGTAGACGAAAGGGGAGGTTATGACCCACCCACATACATCAGGGACCTCTCCATCCATCATACATTCTCTCGCTGCGTCACAGTCCATGGCTCCAATGGCTTGTTGATCAAGGACGTTGTGGGCTATAACTCTTTGGGCCACTGCTTCTTCACGGAAGATGGGCCGGAGGAACGCAACACTTTTGACCACTGTCTTGGCCTCCTTGTCAAGTCTGGAACCCTCCTCCCCTCGGACCGTGACAGCAAGATGTGCAAGATGATCACAGAGGACTCCTACCCGGGGTACATCCCCAAGCCCAGGCAAGACTGCAATGCTGTGTCCACCTTCTGGATGGCCAATCCCAACAACAACCTCATCAACTGTGCCGCTGCAGGATCTGAGGAAACTGGATTTTGGTTTATTTTTCACCACGTACCAACGGGCCCCTCCGTGGGAATGTACTCCCCAGGTTATTCAGAGCACATTCCACTGGGAAAATTCTATAACAACCGAGCACATTCCAACTACCGGGCTGGCATGATCATAGACAACGGAGTCAAAACCACCGAGGCCTCTGCCAAGGACAAGCGGCCGTTCCTCTCAATCATCTCTGCCAGATACAGCCCTCACCAGGACGCCGACCCGCTGAAGCCCCGGGAGCCGGCCATCATCAGACACTTCATTGCCTACAAGAACCAGGACCACGGGGCCTGGCTGCGCGGCGGGGATGTGTGGCTGGACAGCTGCCGGTTTGCTGACAATGGCATTGGCCTGACCCTGGCCAGTGGTGGAACCTTCCCGTATGACGACGGCTCCAAGCAAGAGATAAAGAACAGCTTGTTTGTTGGCGAGAGTGGCAACGTGGGGACGGAAATGATGGACAATAGGATCTGGGGCCCTGGCGGCTTGGACCATAGCGGAAGGACCCTCCCTATAGGCCAGAATTTTCCAATTAGAGGAATTCAGTTATATGATGGCCCCATCAACATCCAAAACTGCACTTTCCGAAAGTTTGTGGCCCTGGAGGGCCGGCACACCAGCGCCCTGGCCTTCCGCCTGAATAATGCCTGGCAGAGCTGCCCCCATAACAACGTGACCGGCATTGCCTTTGAGGACGTTCCGATTACTTCCAGAGTGTTCTTCGGAGAGCCTGGGCCCTGGTTCAACCAGCTGGACATGGATGGGGATAAGACATCTGTGTTCCATGACGTCGACGGCTCCGTGTCCGAGTACCCTGGCTCCTACCTCACGAAGAATGACAACTGGCTGGTCCGGCACCCAGACTGCATCAATGTTCCCGACTGGAGAGGGGCCATTTGCAGTGGGTGCTATGCACAGATGTACATTCAAGCCTACAAGACCAGTAACCTGCGAATGAAGATCATCAAGAATGACTTCCCCAGCCACCCTCTTTACCTGGAGGGGGCGCTCACCAGGAGCACCCATTACCAGCAATACCAACCGGTTGTCACCCTGCAGAAGGGCTACACCATCCACTGGGACCAGACGGCCCCCGCCGAACTCGCCATCTGGCTCATCAACTTCAACAAGGGCGACTGGATCCGAGTGGGGCTCTGCTACCCGCGAGGCACCACATTCTCCATCCTCTCGGATGTTCACAATCGCCTGCTGAAGCAAACGTCCAAGACGGGCGTCTTCGTGAGGACCTTGCAGATGGACAAAGTGGAGCAGAGCTACCCTGGCAGGAGCCACTACTACTGGGACGAGGACTCAGGGCTGTTGTTCCTGAAGCTGAAAGCTCAGAACGAGAGAGAGAAGTTTGCTTTCTGCTCCATGAAAGGCTGTGAGAGGATAAAGATTAAAGCTCTGATTCCAAAGAACGCAGGCGTCAGTGACTGCACAGCCACAGCTTACCCCAAGTTCACCGAGAGGGCTGTCGTAGACGTGCCGATGCCCAAGAAGCTCTTTGGTTCTCAGCTGAAAACAAAGGACCATTTCTTGGAGGTGAAGATGGAGAGTTCCAAGCAGCACTTCTTCCACCTCTGGAACGACTTCGCTTACATTGAAGTGGATGGGAAGAAGTACCCCAGTTCGGAGGATGGCATCCAGGTGGTGGTGATTGACGGGAACCAAGGGCGCGTGGTGAGCCACACGAGCTTCAGGAACTCCATTCTGCAAGGCATACCATGGCAGCTTTTCAACTATGTGGCGACCATCCCTGACAATTCCATAGTGCTTATGGCATCAAAGGGAAGATACGTCTCCAGAGGCCCATGGACCAGAGTGCTGGAAAAGCTTGGGGCAGACAGGGGTCTCAAGTTGAAAGAGCAAATGGCATTCGTTGGCTTCAAAGGCAGCTTCCGGCCCATCTGGGTGACACTGGACACTGAGGATCACAAAGCCAAAATCTTCCAAGTTGTGCCCATCCCTGTGGTGAAGAAGAAGAAGTTGGGTATGGACTACAAGGATGACGATGACAAGGATTACAAAGACGACGATGATAAGGACTATAAGGATGATGACGACAAATGAGCTAGCACATAACTTACGGTAAATGGCCCGCCTGGCTGACCGCCCAACGACCCCCGCCCATTGACGTCAATAGTAACGCCAATAGGGACTTTCCATTGACGTCAATGGGTGGAGTATTTACGGTAAACTGCCCACTTGGCAGTACATCAA
