# Supplementary figures and images for: Inhibition of CEMIP potentiates the effect of sorafenib on metastatic hepatocellular carcinoma by reducing the stiffness of lung metastases
Source: Cell Death Dis. 2023 Jan 13;14(1):25. doi: 10.1038/s41419-023-05550-4 (PMC9839779; doi:10.1038/s41419-023-05550-4)

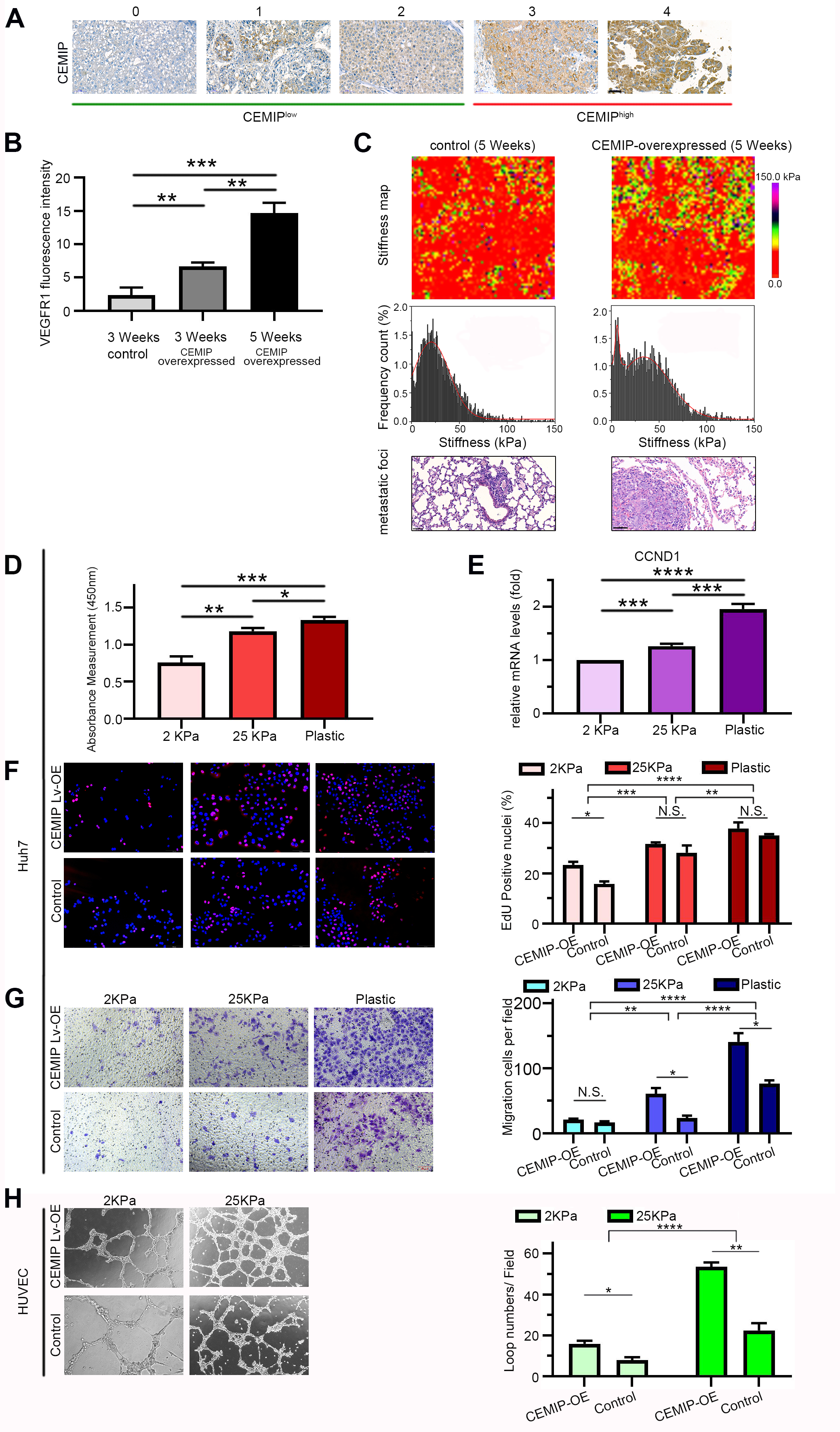

Supplement: Supplementary file 3 — Supplementary Figure 1 [file 41419_2023_5550_MOESM3_ESM.tif]

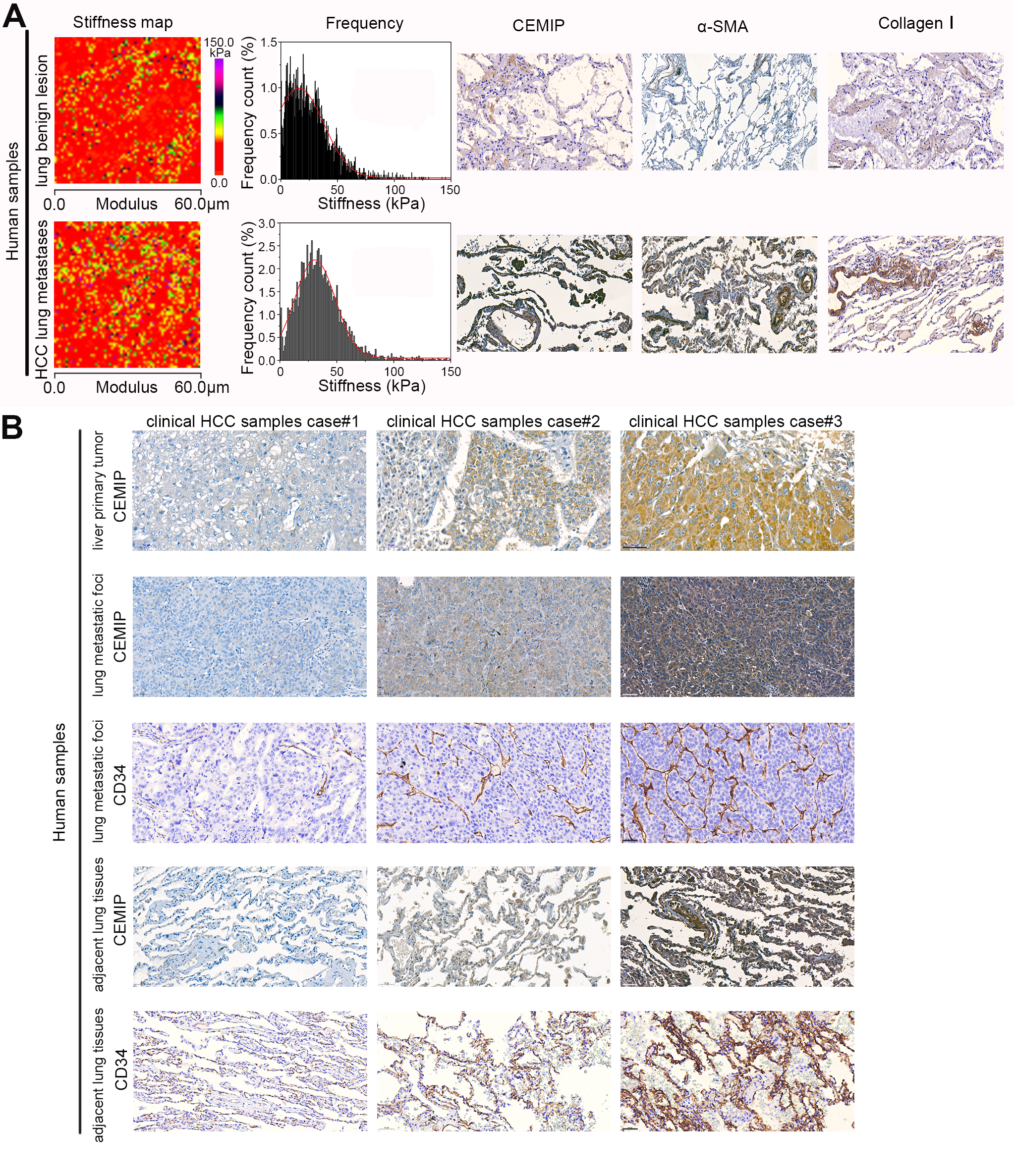

Supplement: Supplementary file 4 — Supplementary Figure 2 [file 41419_2023_5550_MOESM4_ESM.tif]

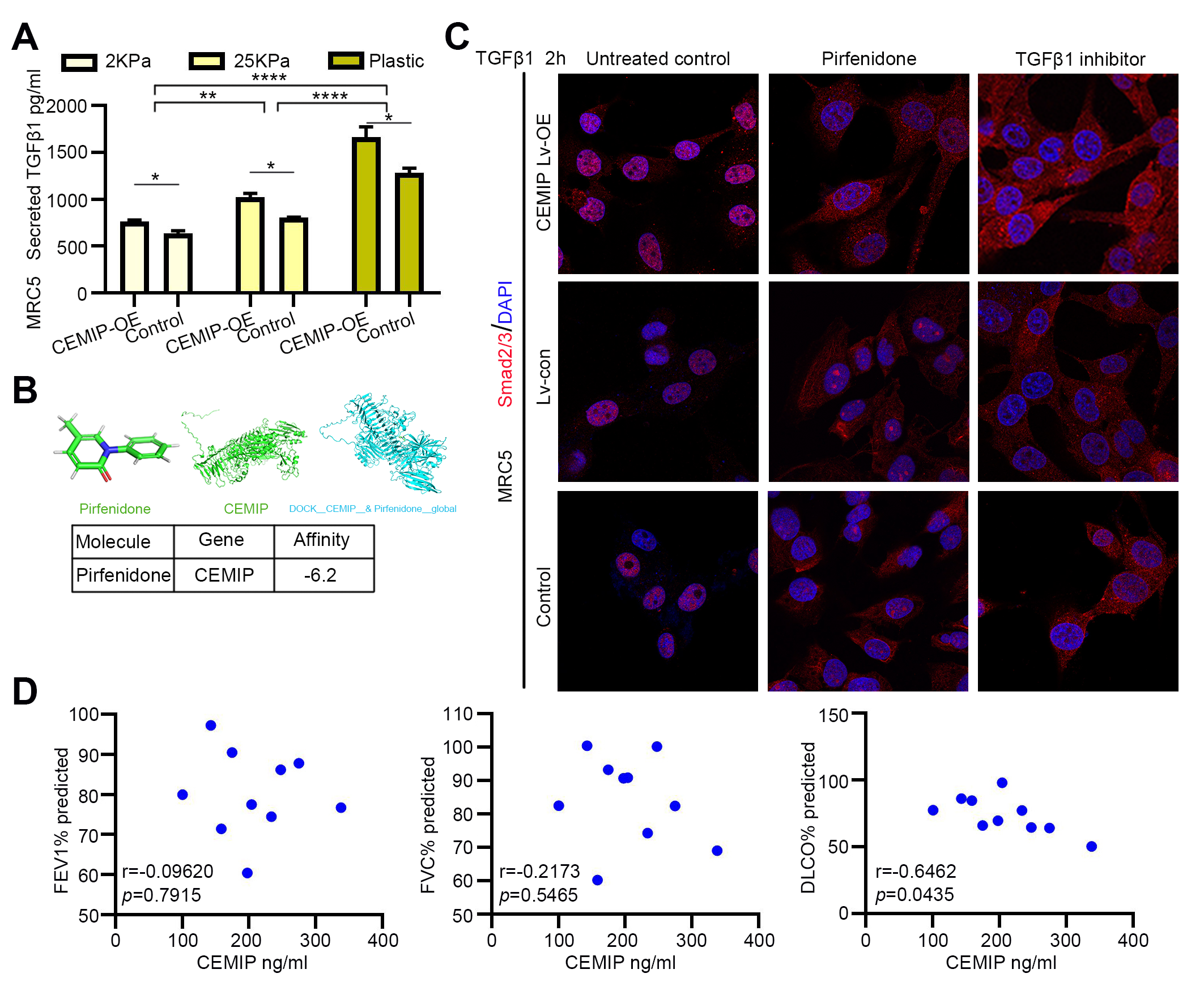

Supplement: Supplementary file 5 — Supplementary Figure 3 [file 41419_2023_5550_MOESM5_ESM.tif]

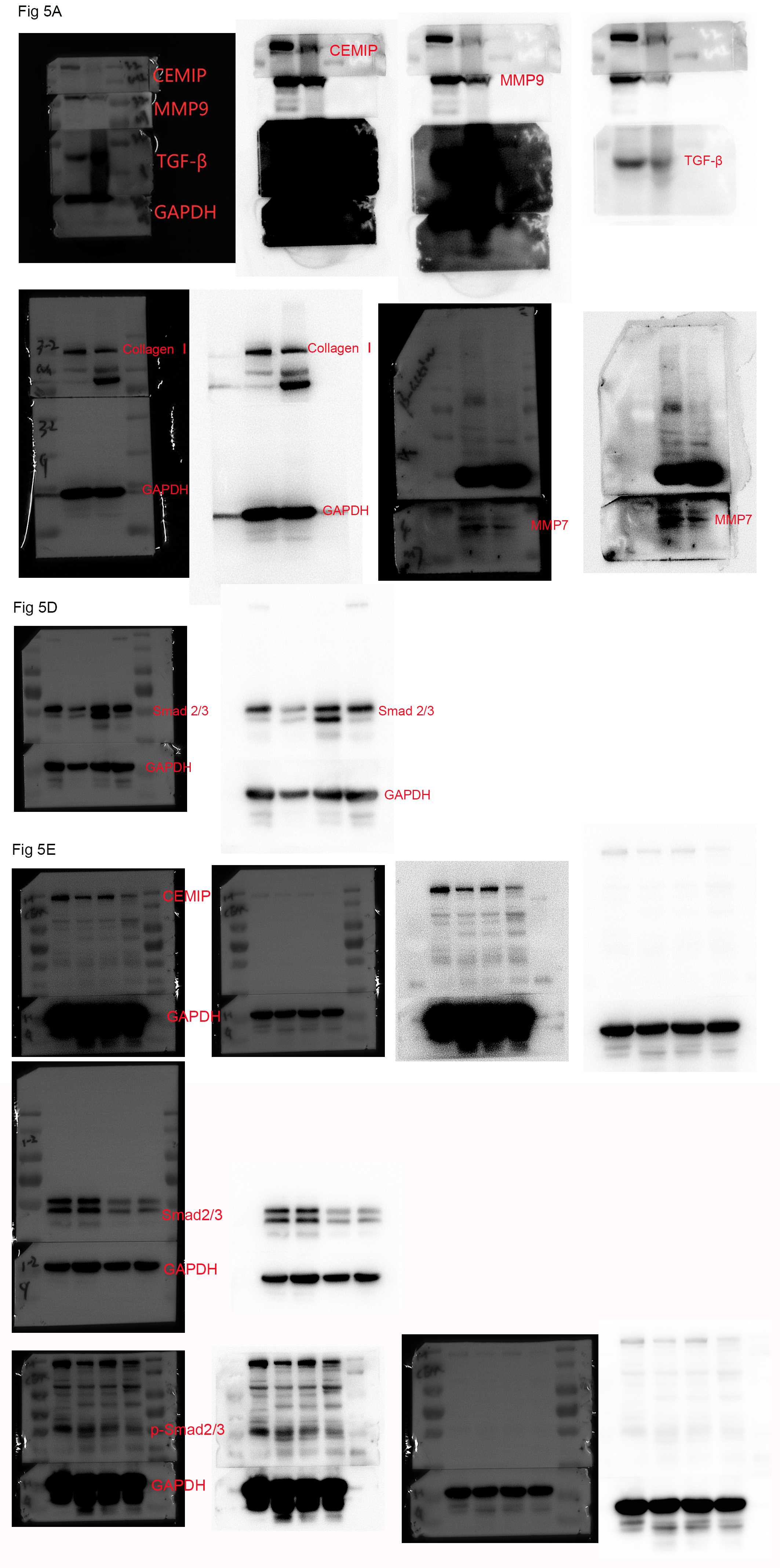

Supplement: Supplementary file 6 — Original Data File [file 41419_2023_5550_MOESM6_ESM.tif]
